# Comparative Analyses Identify the Contributions of Exotic Donors to Disease Resistance in a Barley Experimental Population

**DOI:** 10.1534/g3.113.007294

**Published:** 2013-11-01

**Authors:** Zhou Fang, Amber Eule-Nashoba, Carol Powers, Thomas Y. Kono, Shohei Takuno, Peter L. Morrell, Kevin P. Smith

**Affiliations:** * Department of Agronomy and Plant Genetics, University of Minnesota, St. Paul, Minnesota 55108; † Graduate University for Advanced Studies, Hayama, Kanagawa 240-0193, Japan

**Keywords:** disease resistance, introgression, allele frequency, identity-by-state, breeding

## Abstract

Introgression of novel genetic variation into breeding populations is frequently required to facilitate response to new abiotic or biotic pressure. This is particularly true for the introduction of host pathogen resistance in plant breeding. However, the number and genomic location of loci contributed by donor parents are often unknown, complicating efforts to recover desired agronomic phenotypes. We examined allele frequency differentiation in an experimental barley breeding population subject to introgression and subsequent selection for *Fusarium* head blight resistance. Allele frequency differentiation between the experimental population and the base population identified three primary genomic regions putatively subject to selection for resistance. All three genomic regions have been previously identified by quantitative trait locus (QTL) and association mapping. Based on the degree of identity-by-state relative to donor parents, putative donors of resistance alleles were also identified. The successful application of comparative population genetic approaches in this barley breeding experiment suggests that the approach could be applied to other breeding populations that have undergone defined breeding and selection histories, with the potential to provide valuable information for genetic improvement.

Experimental evolution studies have long been valued as a means of gaining insight into the rate and degree of response to selection (Burke and Rose 2009). Recent studies have demonstrated that when combined with single-nucleotide polymorphism (SNP) genotyping or resequencing and comparative population genetic approaches, experimental populations can provide a rapid means of identification of loci responding to selection (Burke *et al.* 2010; Turner *et al.* 2011; Orozco-Terwengel *et al.* 2012). The approaches have been successfully applied to a number of systems, from experimental populations of microbes exposed to high-temperature regimes (Tenaillon *et al.* 2012) to *Drosophila* populations selected for longevity (Teotónio *et al.* 2009; Burke *et al.* 2010). These studies demonstrate the feasibility of identifying adaptive mutations in experimental populations.

Although plant breeding populations are themselves highly successful long-term experimental evolution studies, there is a long-standing tradition of developing experimental populations to supplement traditional breeding approaches (Harlan and Martini 1929; Suneson 1956; Dudley and Lambert 2004). These experimental populations explore both the potential for local adaptation (Allard *et al.* 1972) and the genetic basis of response to selection (Clegg *et al.* 1972; Weir *et al.* 1972). Experimental plant populations have also been used to test the role of genetic incompatibility in the formation of hybrid species (Rieseberg *et al.* 1996) and the potential for trait introgression in cultivated species (Lin *et al.* 1995; Jiang *et al.* 2000).

Historically, uses of population genetic approaches to identify genetic changes in experimental populations have been limited to a few markers, limiting inference regarding the loci contributing to genetic differentiation to single markers representing large genomic regions (Allard *et al.* 1972; Rieseberg *et al.* 1996). Recently, the availability of high-density SNP genotyping or resequencing data has provided the potential to identify precise genomic regions that have been undergoing selection. Strong directional selection has the potential to produce populations with dramatic differentiation in allele frequency, a potential signal of selection (Lewontin and Krakauer 1973), which could be detectable either by SNP genotyping (Teotónio *et al.* 2009) or by resequencing (Burke *et al.* 2010; Turner *et al.* 2011). Patterns of linkage disequilibrium (LD) (Sabeti *et al.* 2002) or changes in patterns of identity-by-state (IBS) (Albrechtsen *et al.* 2010) can also suggest recent selection.

In the present study, we report a population genetic examination of an experimental barley breeding population developed in response to epidemic levels of the fungal pathogen *Fusarium graminearum*, the causal agent of *Fusarium* head blight (FHB). The prevalence and spread of this pathogen increased in the Midwestern United States in the early 1990s (McMullen *et al.* 1997) and revealed limited genetic variation for disease resistance among existing barley cultivars and the need to introduce novel variation for resistance into breeding programs. During the 35 years preceding the FHB outbreak, the University of Minnesota barley breeding program made use of relatively closed pedigrees in an advanced cycle breeding scheme (Rasmusson and Phillips 1997) that primarily focused on crosses among elite lines from within the breeding population (Condón *et al.* 2009). After the outbreak, numerous exotic sources of elite lines with known FHB resistance and reduced deoxynivalenol (DON) mycotoxin (which is produced by the pathogen) concentration were evaluated and introduced as parents in the breeding population to enhance FHB resistance (Smith *et al.* 2013).

Novel adaptive mutations, including resistance to a pathogen, are likely to be rare in breeding populations, because mutation frequency is dependent on effective population size. Introduction of resistance alleles from standing variation outside the breeding population is the primary mechanism to increase disease resistance (Fetch *et al.* 2003). We report the genetic effects of introgression from a diverse set of 13 barley lines carrying FHB resistance into an existing breeding population. The immediate goal of this experimental population (hereafter referred to as the Reopened population) is to provide substantial improvement in resistance to FHB infection. The Reopened population was compared with a contemporaneous sample of breeding lines from the primary Minnesota breeding population, never subject to introgression of FHB-resistant parents and maintained under a closed pedigree (hereafter referred to as the Closed population) permitting identification of loci potentially subject to selection for FHB resistance in the Reopened population. We demonstrate that comparative population genetic approaches applied to this experimental population provide a complementary approach to QTL and association mapping methods for identifying loci that underlie important phenotypes.

## Materials and Methods

### Plant materials

**Figure 1:**
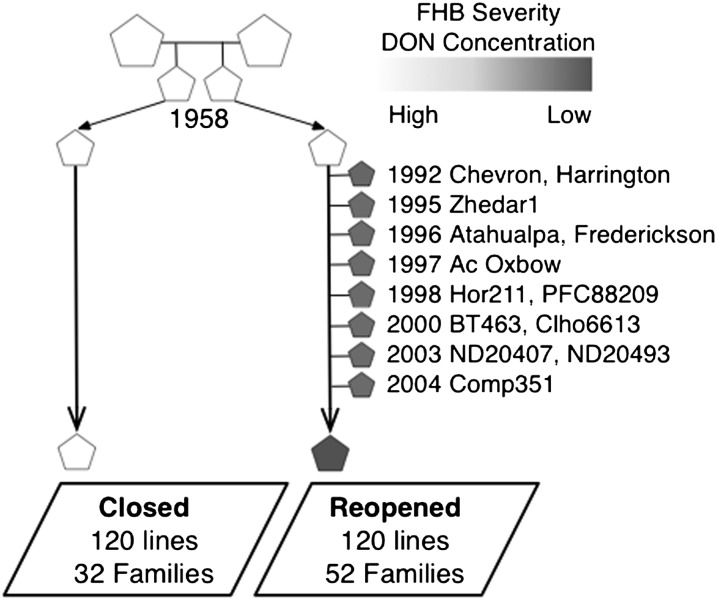
Breeding history of the Closed and Reopened populations. Filled shapes designate individuals carrying *Fusarium* head blight (FHB) resistance and/or reduced deoxynivalenol (DON) accumulation. The Reopened population acquired reduced FHB severity and DON concentration through the introgression from 13 donor lines between 1992 and 2004.

Breeding lines for this study were derived from the six-row malting barley breeding program at the University of Minnesota, which began in the early 1900s. The genetic base of this breeding population is quite narrow, with ∼50% of the six-row germplasm in North America tracing to five ancestors (Martin *et al.* 1991). In the early 1990s, the original advanced cycle breeding strategy was maintained for part of the breeding program while a new strategy that introduced exotic sources of FHB resistance was implemented in parallel. This resulted in two parallel breeding populations within the breeding program with different breeding histories. To compare these two populations, we created two panels of 120 breeding lines that were representative of the two populations. The Closed panel comprises lines from the advanced cycle breeding program (elite × elite) with a relatively closed pedigree, *i.e.*, few new founders were introduced after 1958 when the strategy was initiated (Condón *et al.* 2008; Condón *et al.* 2009). The Reopened panel comprises lines from families derived from the introduction of 13 new donors to the Closed population in response to the FHB epidemic (Figure 1). The lines in each panel were selected from a period of transition between advanced cycle breeding and introduction of disease-resistant parents (2003–2007), such that we could adequately sample both breeding populations. Lines in each panel were selected to maximize the number of families represented within each population; the Closed panel sampled 32 (94%) of 34 families in the Closed population that advanced to preliminary yield trials, and the Reopened panel sampled 52 (87%) of 60 families in the Reopened population. Lines selected for both panels were based on seed or DNA availability, with preference given to lines with malting quality data.

The typical development of breeding lines begins with a cross between two parents in the fall followed by self-pollination of the F_1_ in a greenhouse in the winter. Parents were typically selected after they had been evaluated for 2 years in yield trials based on agronomic performance and acceptable malting quality for the Closed population, and additionally for FHB resistance in the Reopened population. F_2_ plants were grown in the field and advanced by single-seed descent to the F_4_ generation without selection. In the second summer after the initial cross, selection was imposed on the F_4:5_ lines. For the Closed population, F_4:5_ lines were planted in unreplicated single row plots at a single location and visually selected based on general agronomic traits, including maturity, heading date (flowering time), plant height, stem breakage, lodging, and plump kernels. For the Reopened population, F_4:5_ lines were planted in single-row plots at two disease nurseries with two replicates and evaluated for FHB disease severity. For lines selected with low levels of disease, harvested grain from each plot was analyzed for DON produced by the pathogen. Further selection was imposed for low DON concentration in the grain. In both the Closed and Reopened populations, ∼10% selection was imposed. The selected lines were advanced to preliminary yield trials in the third summer after the cross. More detailed information on the experimental population can be found in Smith *et al.* (2010) and Smith *et al.* (2013).

### DNA extraction and genotyping

DNA was extracted from a single F_4:6_ seedling from each breeding line and from a bulk of five or more seedlings for each of the exotic donor lines using the CTAB and chloroform method (Sambrook *et al.* 1989). The 1536 SNPs assayed here [barley oligonucleotide pool assay 1 (BOPA1)] were identified based on Sanger resequencing of expressed sequence tags, where Morex (an important historical Minnesota cultivar) is the most frequently represented genotype (Close *et al.* 2009). Genotyping was conducted at the USDA-ARS Regional Small Grains Genotyping Laboratory at Fargo, North Dakota. All lines were genotyped with BOPA1 SNPs using Illumina GoldenGate technology (Illumina, San Diego, CA). Genotypes were called using the Illumina Beadstation software. Eleven of the 13 donor lines were genotyped. Genotype and pedigree data from the Closed and Reopened panels are available in The Triticeae Toolbox (http://triticeaetoolbox.org/), an updated version of The Hordeum Toolbox (Blake *et al.* 2012). Genotypic and phenotypic data for individual lines are available for download by selecting populations labeled “MN Reopened” and “MN Closed” under “select lines by properties.”

### Data analysis

As a part of SNP data quality control, all SNPs monomorphic in the combined Closed and Reopened panels were removed. We also removed SNPs and individual samples with ≥10% missing data or with ≥10% observed heterozygosity. Barley is a selfing species and progeny from breeding crosses were genotyped at the F_4:6_ generation, so SNPs or samples with elevated heterozygosity are likely attributable to genotyping errors. SNP positions were based on the consensus genetic map of Muñoz-Amatriaín *et al.* (2011) and are depicted in Supporting Information, Figure S1.

SNPs were annotated to determine the genes of origin. Annotations were performed using the SNP annotation tool, SNPMeta (T. Y. Kono, K. Seth, J. A. Poland, and P. L. Morrell, in press) based on the contextual sequence used for the Illumina SNP assay design (Close *et al.* 2009). SNP contextual sequences were used as BLAST queries against the NCBI nucleotide (nt) database. The best BLAST hit with an annotated coding sequence was downloaded and aligned to the SNP contextual sequence. Information, including gene name and whether the SNP causes a synonymous or nonsynonymous change, was recorded for each SNP. The majority of annotations originate from a large collection of full-length cDNAs (Sato *et al.* 2009; Matsumoto *et al.* 2011). The number of annotated barley genes within genomic regions identified in our studies was inferred using relative genetic map positions from the barley GenomeZipper (Mayer *et al.* 2011).

LD measured as *r^2^* (correlation coefficient) (Hill and Robertson 1968) for all possible pairwise comparisons on each linkage group was calculated in R (R Development Core Team 2011), and the R package LDheatmap (Shin *et al.* 2006) was used to generate plots of LD relative to genetic distance. The package hierfstat (Goudet 2005) was used to calculate haploid *F*_ST_ for each SNP based on comparison of the Closed and Reopened panels, with heterozygous SNPs treated as missing data. An empirical threshold of the top 2.5% of *F*_ST_ values on a per-SNP basis was used to identify *F*_ST_ values that differed dramatically from the genome-wide average. The R package ape (Paradis *et al.* 2004) was used to calculate percent pairwise difference between the Closed or Reopened panels and donor lines. Other SNP descriptive statistics were calculated using the programs compute and sharedPoly from the libsequence C++ library (Thornton 2003), including number of segregating sites, number of singletons, and mean per-SNP pairwise diversity (Tajima 1983) within each of the Ancestral, Closed, and Reopened panels, and number of private and shared SNPs.

Segments of IBS were identified using GERMLINE (Gusev *et al.* 2009). Each SNP and a minimum of five adjacent SNPs were considered sequentially, with length of shared haplotypes extended until mismatch. IBS was calculated based on comparison of each of the Reopened lines and their respective donor or donors (Table S1). On each linkage group, we jointly considered all IBS segments based on each donor line, thus there were many overlapping IBS segments along the linkage group for each donor line. The number of IBS segments at each SNP was determined by summing over the number of lines in the Reopened panel that included an IBS segment for each SNP.

### Simulation

To determine if patterns of allele frequency differentiation between the Closed and Reopened patterns could occur in the absence of selection, we performed coalescent simulation implemented in the program ms (Hudson 2002) (see details in File S1). An initial set of simulations was focused on differentiation between the Ancestral population (donor lines) and the MN breeding population, which forms the basis of the Closed panel (Figure S2). The Ancestral and Closed panels were each represented by 120 chromosomes. The Ancestral panel includes the 11 donor parents genotyped in this study supplemented with 109 lines chosen at random from parents for the barley nested association mapping (NAM) population to balance the panel size to the same as the Closed and Reopened panels and to better-represent the donor population. The NAM parents are a randomly chosen sample of USDA National Small Grains Collection and therefore represent the diverse panel of cultivated barley lines serving as a source for the donor population.

The folded site frequency spectrum (SFS) for the genotyped SNPs is skewed toward common variants (Close *et al.* 2009) (Figure S3A). To simulate ascertainment bias, we used a custom Python script that conditions on a discovery panel of fixed size and a minimum minor allele frequency for samples in the discovery panel. The first *n* chromosomes in the simulation were designated as the discovery panel, and sites that had a minor allele frequency below a user-defined threshold in the discovery panel were removed from the simulation dataset. If migration matrices were specified, then the script assumed that the first population listed was the population in which discovery was performed. Thus, SFS in simulation reflects what is observed in the empirical data.

The mutation parameter θ = *4N*_0_μ and crossover rate parameter ρ = *4N_0_r*, where *N*_0_ is the effective population size of the ancestral population, were adjusted to reflect observed values of percent pairwise diversity and levels of LD (mean *r^2^*) calculated using tools from the libsequence library (Thornton 2003) for each linkage group. We simulated a bottleneck in the establishment of the Closed panel that started at time T_1_ and ended at T_2_ (Figure S2). Time T_1_ was set to 8000 generations before present, when barley began to be disseminated from Western Asia (Pinhasi *et al.* 2005; Pinhasi *et al.* 2012), and T_2_ varied over a uniform distribution of 15 to 8000 generations U(15,8000). The Reopened panel started ∼15 generations ago (T_3_). The relative size of the Closed panel is a proportion of the donor population U(0,0.02). We refined this uniform interval for the relative size based on initial simulations.

To determine the likely time of the end of the bottleneck (T_2_) and the relative size of the Closed panel, we performed one million simulations and compared the simulated and observed values of pairwise diversity (*P*_S_ and *P*_O_) in the Closed panel. Using rejection sampling, we retained simulations if |*P*_S_ − *P*_O_|/*P*_O_ < ε. After preliminary survey, we chose the acceptance rate of ε = 0.2 and confirmed that any choice of ε did not affect the result (not shown). The end of the bottleneck (T_2_) and the relative size were determined by averaging these simulations.

To determine the expected distribution for *F*_ST_ values between the Closed panel and the Reopened panel based on a neutral demographic scenario involving only migration and introgression, we compared *P*_S_ and *P*_O_ in the Reopened panel to estimate the migration rate from the donor lines to the Reopened panel. The previous distribution of the migration rate was sampled from U(0,10,000). The migration rate is based on *4N_0_m*. We retained simulations if |*P*_S_ − *P*_O_|/*P*_O_ < ε. The most likely migration rate was determined by averaging these simulations. Using the most likely migration rate from the donor lines to the Reopened panel, we simulated the complete population history and calculated *F*_ST_ between the simulated Closed and Reopened panels using 100,000 simulations.

## Results

### Summary statistics for the Closed and Reopened panels

Quality control resulted in a data set with 990 SNPs in 237 lines. This included the elimination of 546 SNPs, 465 of which were monomorphic in both panels. We also eliminated two samples in the Closed panel and one sample in the Reopened panel because of SNP genotype quality. The average observed heterozygosity across the complete data set was 0.43%. There were 54 SNPs private to the Closed panel compared with 482 SNPs private to the Reopened panel (Table 1). There were 478 SNPs and 895 SNPs that were shared between the donor lines and the Closed and Reopened panels, respectively (Table 1). Pairwise diversity in the Closed panel was 0.15 genome-wide compared with 0.16 in the Reopened panel, indicating greater similarity among lines in the Closed panel (Table 1).

**Table 1:** Summary statistics for lines in the Closed and Reopened panels

Panel	Sample	Segregating Sites	Singletons	Mean *F*_IS_	Mean Pairwise Diversity	Percent Pairwise Difference with Donor Lines (SD)	Private SNPs	Shared SNPs	Shared SNPs with Donor Lines
Ancestral	120	933	11	0.997	0.41	—	—	—	—
Closed	118	502	168	0.945	0.15	0.084 (0.163)	54	448	478
Reopened	119	930	112	0.959	0.16	0.137 (0.130)	482		895

The summary statistics reported include sample size, number of segregating sites, number of singletons, average *F*_IS_, mean pairwise diversity, percent pairwise difference with donor lines, number of private SNPs in each population, and number of shared SNPs in each comparison. Shared SNPs refers to the comparison of the Closed and Reopened panels. SNP, single-nucleotide polymorphism.

### Allele frequency differences between the Closed and Reopened panels

**Figure 2:**
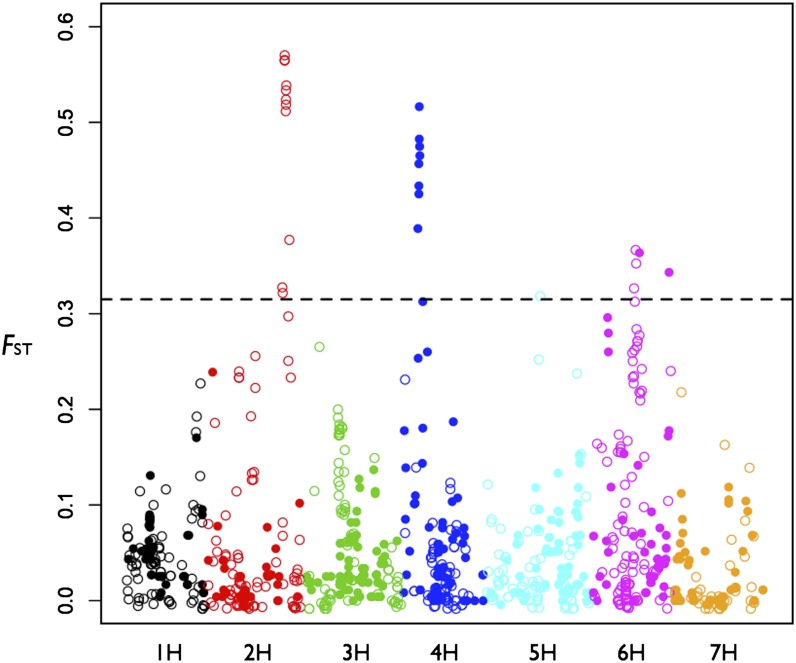
Genome-wide *F*_ST_ plot. The horizontal dashed line is the 97.5^th^ percentile. Colors correspond to linkage groups. Single-nucleotide polymorphisms (SNPs) that are monomorphic in the Closed panel are represented by ● and SNPs that are polymorphic in the Closed panel are represented by ○.

Genome-wide *F*_ST_ averaged 0.057 between the Closed and Reopened panels. Three genomic regions with *F*_ST_ values exceeding the 97.5^th^ percentile (*F*_ST_ ≥ 0.315) were identified on linkage groups 2H, 4H, and 6H (Figure 2). Also, a single SNP on 5H exceeded the *F*_ST_ threshold. In the high *F*_ST_ regions on 2H and 4H, the majority of SNPs (9 out of 11 SNPs on 2H and 8 out of 9 SNPs on 4H) were above the 97.5^th^ percentile threshold (Table 2). On 6H, only four of 20 SNPs >∼10 cM high *F*_ST_ region exceed the 97.5^th^ percentile threshold.

**Table 2:** Single-nucleotide polymorphisms in the high *F*_ST_ regions on linkage group 2H, 4H, 5H, and 6H

LG	SNP	GenBank ID	cM	*F*_ST_	Frequency Donor	Frequency Closed	Frequency Reopened
2H	11_10446	XM_003560174	140.69	0.57	0.73	0.07	0.72
	11_20480	AY162186	140.69	0.57	0.91	0.08	0.72
	11_21440	AK360366	140.69	0.56	0.91	0.08	0.73
	11_21406	AK370573	142.67	0.51	0.73	0.10	0.72
	11_21370	AK362193	143.18	0.52	0.73	0.11	0.74
	11_11486	X58138	143.18	0.52	0.64	0.10	0.73
	11_21459	AM039897	143.18	0.53	0.64	0.10	0.73
	11_10109	AK362400	143.71	0.54	0.55	0.11	0.73
	11_10065	AK376660	147.37	0.30	0.64	0.59	0.15
	11_20215	AK371957	147.37	0.25	0.64	0.59	0.15
	11_20895	AK366193	149.27	0.38	0.55	0.64	0.17
4H	11_10132	AK358845	26.20	0.39	0.55	1.00	0.60
	11_20210	AK375913	26.71	0.25	0.73	1.00	0.74
	11_20422	XM_003560743	28.00	0.46	0.64	1.00	0.53
	11_21070	AK355304	28.00	0.43	0.64	1.00	0.55
	11_20302	AK372064	28.00	0.43	0.55	1.00	0.56
	11_20680	—	28.75	0.48	0.55	0.00	0.50
	11_21418	X62388	28.75	0.52	0.91	0.00	0.53
	11_20109	AK360657	29.34	0.47	0.91	0.00	0.49
	11_20777	AK364484	29.76	0.47	0.82	0.00	0.47
5H	11_21321	AK369180	97.49	0.32	0.91	0.01	0.37
6H	11_10040	AK357672	74.65	0.00	0.64	1.00	0.99
	11_10124	AK367485	74.65	0.04	0.73	0.98	0.90
	11_20015	X95863	74.65	0.33	0.91	0.03	0.42
	11_20709	AK363507	74.65	0.23	0.73	0.03	0.33
	11_20714	AK354049	75.28	0.26	0.64	0.99	0.71
	11_20468	AK362215	76.03	0.00	0.73	0.99	1.00
	11_21469	—	76.03	0.31	0.91	0.03	0.41
	11_20636	AK365203	77.53	0.37	0.91	0.01	0.41
	11_11329	AK364583	78.52	0.00	0.91	0.98	0.99
	11_20673	HQ661104	78.52	0.35	0.82	0.01	0.42
	11_20892	AK376359	79.17	0.28	0.73	0.00	0.31
	11_11349	AK361558	80.06	0.27	0.82	0.01	0.31
	11_20784	AK365537	80.06	0.00	0.82	0.99	1.00
	11_11459	AK363684	80.86	0.27	0.64	0.99	0.69
	11_21256	AK359524	80.86	0.27	0.64	0.99	0.69
	11_10469	AK355738	81.79	0.00	0.91	0.99	1.00
	11_20053	AK370710	81.79	0.14	0.64	0.00	0.15
	11_20488	AK358007	84.47	0.36	0.55	1.00	0.63
	11_20682	AY029260	84.47	0.22	0.64	0.01	0.25
	11_20969	—	84.47	0.28	0.82	0.01	0.31
	11_11111	AK366470	139.09	0.34	0.55	1.00	0.63
	11_20687	AK366288	139.09	0.18	0.73	1.00	0.81

Frequency Donor is the frequency of donor alleles (the major alleles in the donor lines). Frequency Closed and Frequency Reopened are the frequency in the Closed and the Reopened panels, respectively, of the donor alleles. LG, linkage group; SNP, single-nucleotide polymorphism; ID, identification.

Comparison of minor allele frequency (MAF) demonstrated that, genome-wide, there were far fewer SNPs segregating in the Closed than the Reopened panels (Figure S1). The three primary regions on 2H, 4H, and 6H with the largest difference in MAF between the Closed and Reopened panels corresponded to the three high *F*_ST_ blocks on these linkage groups. In the Closed panel, all SNPs in the high *F*_ST_ block on 4H were monomorphic, but they were polymorphic in the Reopened panel (Figure 2). *F*_ST_ plotted relative to MAF also showed the three clusters on these three linkage groups and indicated that SNPs with high *F*_ST_ on each linkage group also shared similar MAF (Figure S4).

The majority allele in the donor lines (donor allele) tended to occur at higher frequencies in the Reopened panel than in the Closed panel. In the high *F*_ST_ region on 2H, the frequency of the majority donor allele was substantially higher in the Reopened panel compared to the Closed panel for 8 out of the 11 SNPs (Table 2). The region on 4H was monomorphic in the Closed panel, and donor alleles introduced novel variants to the Reopened panel. All 22 SNPs in the high *F*_ST_ regions on 6H were either monomorphic or had low MAF in the Closed panel but were more polymorphic in the Reopened panel (Table 2).

One feature of the MAF that could not be explained by donor introgression is a region involving 34 SNPs at 64–81 cM on linkage group 3H with ∼0.3 MAF in the Closed panel (Figure S1). There were two primary haplotypes for these 34 SNPs (data not shown).

### Simulation

A discovery panel of eight chromosomes with a minimum minor allele count of three reflected the design parameters of the barley OPAs (Close *et al.* 2009). In simulations of the Ancestral population, this discovery scheme also closely matched the observed SFS (Figure S3) (paired *t*-test p-value = 1).

When including the Closed panel in the model, the median of simulated pairwise diversity along each linkage group in the Ancestral panel was 0.054 (95% CI, 0.42–0.68). In the Closed panel, the median of simulated pairwise diversity was 0.007 and pairwise diversity was zero in 35% of simulations. The pairwise diversity in both the Ancestral panel and the Closed panel provided a close fit to the observed data (Table S2). In our simulations, there was convergence in posterior density of relative size of the Closed panel, which was ∼1% of the Ancestral population (Figure S5). There was a wide interval for the most likely timing of the end of the bottleneck, which varied across the range of prior values. When we plotted the density of these two parameters together, the most likely timing of the end of the bottleneck corresponded to the highest likelihood of the relative size, which was at 0.0015, ∼900 generations ago (Figure S6). The relative size of the bottleneck and duration of the bottleneck were confounded as suggested by a previous study (Eyre-Walker *et al.* 1998).

**Figure 3:**
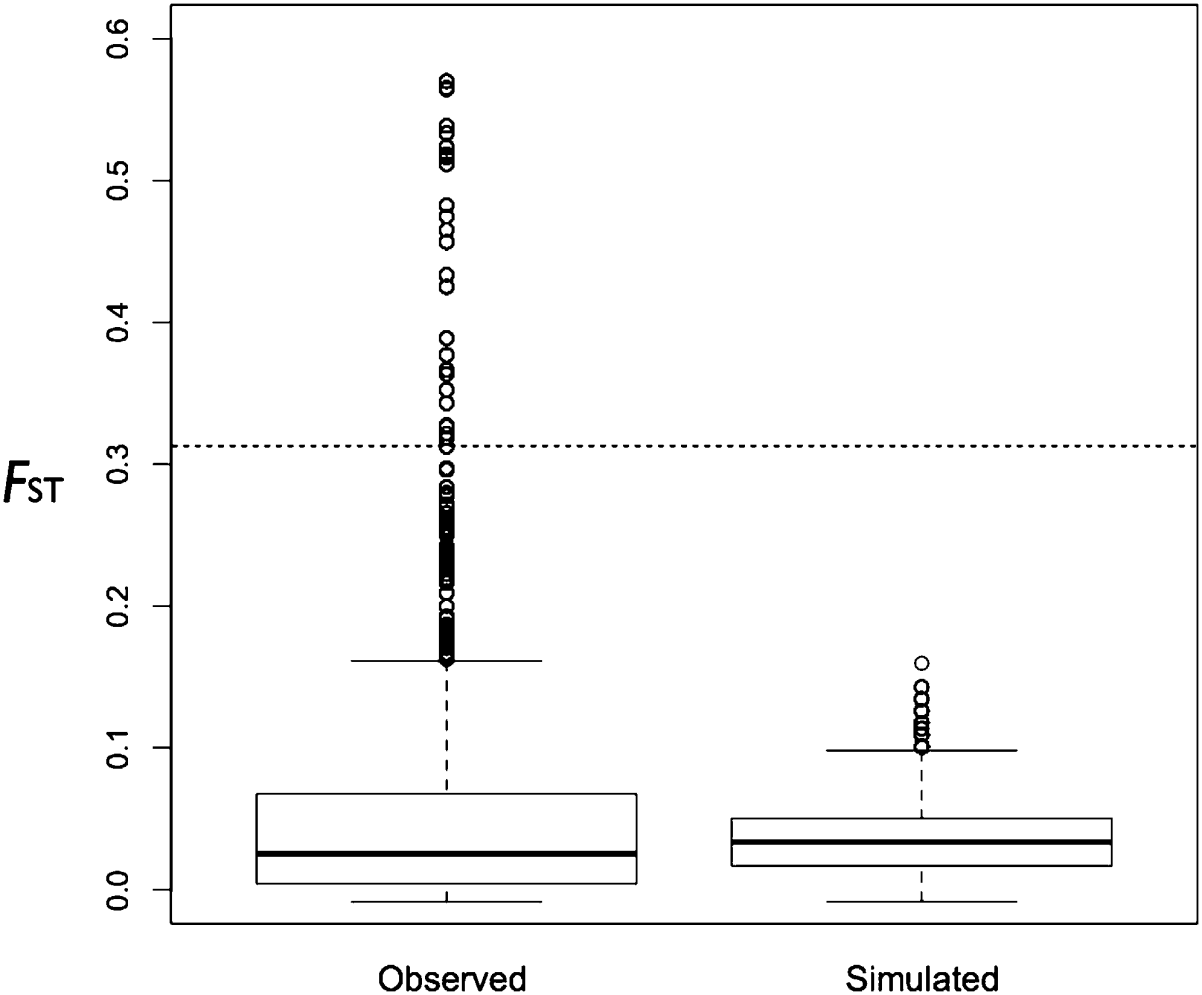
The boxplots for observed and simulated *F*_ST_ between the Closed and Reopened panels. Simulations were based on demography alone. The dashed horizontal line is the 97.5^th^ percentile of *F*_ST_ in the empirical data.

Adding the Reopened panel to the simulation, and simulating migration, the estimated migration rate was *4N_0_m* = 4000, which corresponded to 0.01 migrants per generation over 15 generations (Figure S7). In these simulations, in which demography alone impacted allele frequency (*i.e.*, where we were testing a neutral null hypothesis), the 97.5^th^ percentile of *F*_ST_ was 0.09 between the simulated Closed and Reopened panel compared with *F*_ST_ of 0.315 in the empirical data (Figure 3). This suggests that in the absence of selection, demography alone is unlikely to produce the extreme values of *F*_ST_ observed between the Closed and Reopened panels.

### Segments of IBS

**Figure 4:**
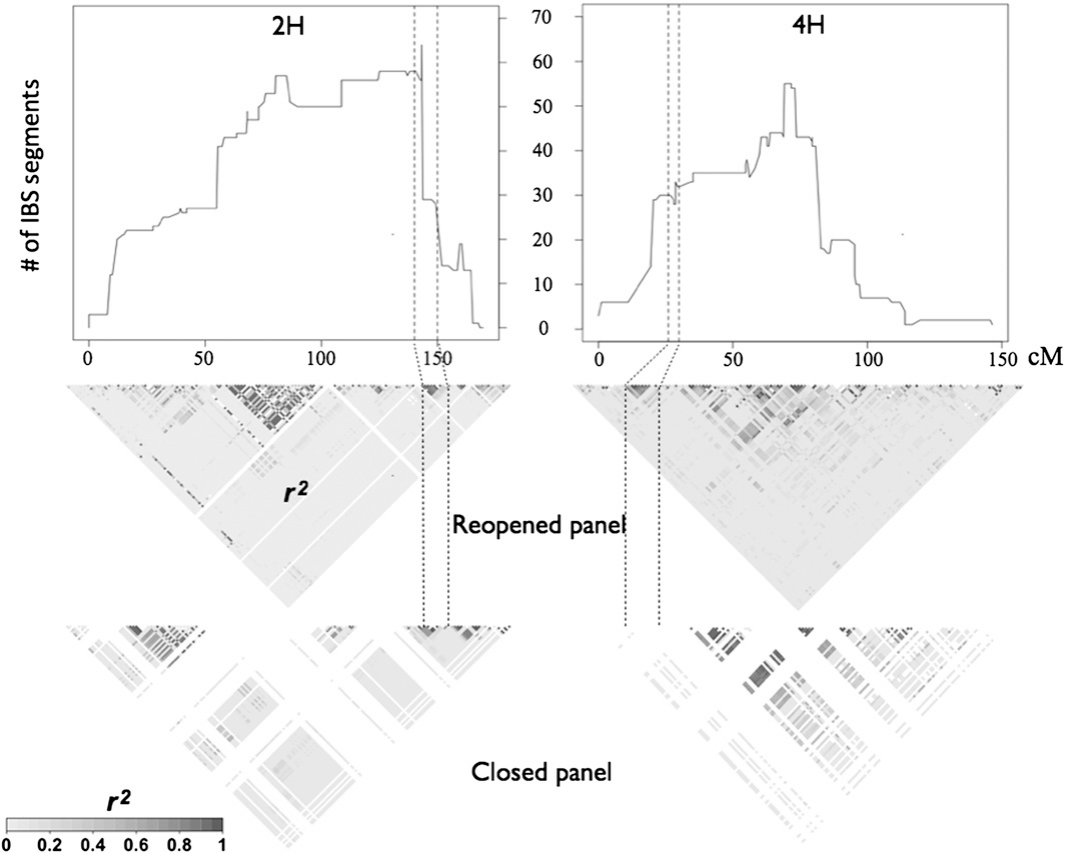
Identity-by-state (IBS) and linkage disequilibrium (LD) plot to linkage groups 2H and 4H. The upper panel shows number of IBS segments between the donor lines and their progeny in the Reopened panel. The vertical dashed lines delimit the high *F*_ST_ block. The middle and lower panels are LD heatmaps of the Reopened and Closed panels, respectively.

**Figure 5:**
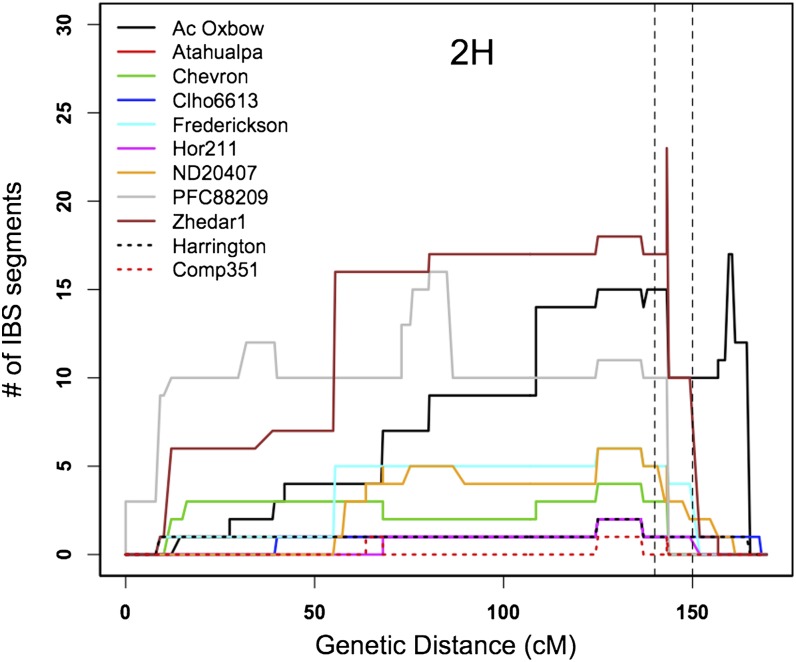
Identity-by state (IBS) between each of the donor lines and their respective progeny in the Reopened panel on 2H. The vertical dashed lines delimit the high *F*_ST_ block.

Individual donors contributed to an average of 12 progeny in the Reopened panel. Zhedar1 contributed to the largest number of progeny, 49, whereas Comp351 and BT463 contributed to only a single individual (Table S1). The highest degree of IBS between the donor lines and their progeny in the Reopened panel on 2H and 6H overlapped the high *F*_ST_ regions (Figure 4 and Figure S8). However, the highest IBS region on 4H did not overlap with the high *F*_ST_ region, but rather it occurred ∼40 cM away (Figure 4). This resulted from a localized contribution of high IBS from donor Hor211 to 24 progeny; excluding this donor, the highest degree of IBS on 4H also overlapped with the high *F*_ST_ region for most donor lines (Figure S9). The degree of IBS declines dramatically at both ends of the chromosome, where SNP number limits the potential to identify long segments that were IBS. Donor line Zhedar1 contributed most to the Reopened panel in the high *F*_ST_ region on 2H (Figure 5), whereas PFC88209 contributed most to the high *F*_ST_ regions on 4H and 6H (Figure S9). We summed the number of IBS segments at each SNP across the genome and across the three major high *F*_ST_ regions. We found little correlation between the timing of introgression of donor lines and the number of IBS segments genome-wide (*r*^2^ = 0.20) as well as in the high *F*_ST_ regions (*r^2^* = 0.10).

### LD in the Closed and Reopened panels

Average genome-wide LD (*r^2^*) among all pairs of SNPs was higher in the Closed panel (0.051) than in the Reopened panel (0.028). LD between adjacent SNPs was also higher in the Closed panel (0.653) compared with the Reopened panel (0.490) (Figure S10).

Blocks of LD were defined as sets of at least three adjacent SNPs that showed greater LD than the median *r*^2^ of adjacent SNPs (0.58). There were 28 blocks in the Closed panel covering a total of 65.13 cM and 45 blocks in the Reopened panel covering a total of 80.63 cM. The average block size in the Closed panel was 2.33 cM, which was greater than that in the Reopened panel (1.79 cM). In the Closed panel, 15.9% of SNPs were in LD blocks, whereas 21.8% of SNPs were in blocks in the Reopened panel.

All the SNPs in the two high *F*_ST_ blocks on linkage groups 2H and 4H were also in high LD (*r^2^* > 0.21, the 97.5^th^ percentile threshold) with each other in the Reopened panel (Figure 4). The LD pattern was less clear in the linkage group 2H block in the Closed panel (*r^2^* = 0.477 *vs.* 0.613) and the SNPs were monomorphic in the linkage group 4H block in the Closed panel (Figure 4). The LD in the high *F*_ST_ region on 6H was similar in the Closed and Reopened panels (*r^2^* = 0.438) (Figure S8).

### Comparison to previous studies

We identified markers from previous studies that occur in genetic map locations adjacent to high *F*_ST_ genomic regions in our comparison (Table S3). The high *F*_ST_ regions on 2H, 4H, and 6H overlapped with the relative genetic map positions of markers associated with DON concentration and FHB resistance in previous QTL mapping studies (Ma *et al.* 2000; Mesfin *et al.* 2003) as well as in a recent GWAS study that included elite breeding lines from four Midwest breeding programs, including the University of Minnesota program (Massman *et al.* 2011).

In addition to SNPs surveyed here (BOPA1), three additional sets of SNPs have been mapped in barley genetic mapping populations (Rostoks *et al.* 2005; Close *et al.* 2009; Muñoz-Amatriaín *et al.* 2011). All BOPA1, BOPA2, pilot oligonucleotide pool assays (POPA), and Scottish Crop Research Institute (SCRI; the SCRI is now known as the James Hutton Institute) SNPs (http://bioinf.hutton.ac.uk/iselect/app/) that fall within annotated genes in the high *F*_ST_ blocks on 2H, 4H, and 6H are listed in Table S4. The genomic regions identified are ∼5 cM (4H) and ∼10 cM (2H and 6H) and include a minimum of 40 (on 4H) or 100 genes (on 2H and 6H).

## Discussion

Several genomic regions putatively subject to selection have been identified using comparative population genetic methods in this barley experimental population. These regions show strong allele frequency differentiation between the Closed and Reopened panels, excess IBS between the Reopened panel and donor lines, and elevated LD in the Reopened panel.

### Variability of allele frequency

In the donor lines Chevron and Frederickson, QTL contributing to FHB resistance have been mapped to the same interval found to have high *F*_ST_ on 2H and 6H (de la Pena *et al.* 1999; Ma *et al.* 2000; Mesfin *et al.* 2003; Massman *et al.* 2011). The SNPs in the two intervals on linkage groups 2H and 4H have *F*_ST_ values in the top 2.5% genome-wide. The plot of *F*_ST_ *vs.* MAF (Figure S4) shows clusters of high *F*_ST_ SNPs on the same linkage group having similar MAF, which suggests these SNPs co-occur, potentially because of selection favoring a relatively small number of haplotypes. Interestingly, all SNPs in the high *F*_ST_ block on 2H are polymorphic within the Closed panel, but SNPs in the high *F*_ST_ block on 4H are monomorphic in the Closed panel (Figure 2 and Table 2). Within the limits of the experiment, this suggests that selection at 4H is more likely to have acted on newly introgressed allelic variation.

In this experimental population, allele frequency changes appear to provide an effective means of identifying genomic regions subject to selection. However, there are limitations to this approach. First, selection on FHB phenotypes is at least partially confounded with selection for agronomically adaptive phenotypes, particularly in the parent used for later generation crosses in the recipient (Reopened) population. Although there are QTL associated with heading date and plant height that are coincident with FHB or DON in the high *F*_ST_ region on 2H and 4H, the QTL associated with heading date and plant height on 6H are not within the high *F*_ST_ regions based on the association mapping study by Massman *et al.* (2011). Second, the family structure within the population may tend to inflate differences in SNP frequency. Third, as with any outlier-based approach, extreme values of *F*_ST_ could result from stochastic processes and thus would represent false-positives when attributed to selection. Finally, in the present study, genetic resolution is limited and differences in allele frequency likely reflect only the general proximity of causative mutations. The level of genetic resolution provided by the high *F*_ST_ regions is comparable to association mapping studies in plant breeding populations (Cockram *et al.* 2010; Massman *et al.* 2011) and is not a major impediment to the utilization of the identified genomic regions in marker-assisted breeding or genomic selection approaches.

### Variability of IBS

Along with assaying allele frequency changes, we used IBS analysis to identify regions that were putatively subject to selection. The high IBS regions on 2H and 6H were within or adjacent to the high *F*_ST_ regions (Figure 4 and Figure S8). The IBS analysis identified a narrower region, because the number of IBS segments at SNP 11_21459 on 2H was much higher than that at both adjacent SNPs. However, the excess IBS region in the high *F*_ST_ region on 4H did not have the highest number of IBS segment on this linkage group (Figure 4), which was primarily contributed by one donor line, Hor211 (Figure S9).

The IBS analysis also has limitations. The timing of introgression of donor lines could influence IBS results, because the lines introgressed recently would have more IBS segments. However, our results show that a strong correlation does not exist between the timing of introgression and the number of IBS segments, and the two donor lines PFC88209 and Zhedar1 that contributed most to the high *F*_ST_ regions were not among the most recently used donors (Figure 1).

### Variability of LD

The distribution and pattern of LD (*r^2^*) differed dramatically between these two panels (Figure 4 and Figure S10). The introduction of allelic diversity to the Reopened panel resulted in a larger number of polymorphic SNPs and a reduction in both average genome-wide and average adjacent SNP LD within this population. Although average LD was lower in the Reopened panel, the LD was higher in the two high *F*_ST_ regions on linkage groups 2H and 4H in the Reopened panel, as can occur in genomic regions subject to recent strong selection (Sabeti *et al.* 2002; McVean 2007) (Figure 4). Therefore, the blocks of LD in the Reopened panel on linkage groups 2H, 4H, and 6H likely resulted from selection on haplotypes for FHB resistance or reduction in DON accumulation. A number of other factors, however, could potentially contribute to localized elevation of LD, including recent admixture (Pfaff *et al.* 2001) or suppressed recombination attributable to chromosomal structural variation (Graubard 1932; Fang *et al.* 2012).

Introducing genetic diversity and shifting selection pressure changes the distribution and pattern of LD among markers and, therefore, between markers and QTL. This suggests that it will be important to use panels of germplasm that are contemporary and relevant to current breeding goals for association analysis and generating prediction models for genomic selection. As genetic distance between two breeding populations increases, the correlation between closely linked markers in the two populations decreases. Based on this, Hamblin *et al.* (2010) cautioned against pooling data from different breeding programs for association analyses. However, similar concerns may apply to populations within a breeding program that have different breeding histories. Recent work evaluating genomic selection prediction accuracy has shown that using a training population from distinct but closely related breeding programs provides less accurate predictions than from a training population representing the target breeding population (Lorenz *et al.* 2012).

## Conclusion

We have used population genetic approaches to identify genomic regions putatively subject to selection subsequent to introgression in a barley breeding experiment. The progenitor-derivative relationship between the two populations in our study is among the simplest possible scenarios for detecting the effects of recent strong selection (Innan and Kim 2008). Other comparative analyses of plant breeding history generally deal with more diverse breeding histories over longer periods of time (Sim *et al.* 2010; van Heerwaarden *et al.* 2012), making inference of the selective pressure on outlier loci more difficult. The identification of the genomic regions previously associated with FHB resistance and DON concentration suggests that the comparative approach applied here is complementary to the identification of trait-associated markers through QTL and association mapping approaches.

There are several advantages to the comparative approach as applied here. The first is the relative speed and minimal expense associated with identification of putatively trait-associated loci. This experiment was conducted within the confines of a breeding program, obviating the need for multiple QTL mapping populations to identify sources of resistance. The program developed two high-yielding malting cultivars from material represented in this population. Rasmusson is representative of the Closed population (Smith *et al.* 2010), whereas Quest (Smith *et al.* 2013), a cultivar with reduced DON accumulation, is derived from donor parents Chevron and Zhedar1 and is a product of the Reopened population. Second, comparison of allele frequencies among populations will remain effective even as divergence and low minor allele frequencies within populations minimize the potential for effective association mapping. Finally, the comparative population genetic approach is also free of *a priori* identification of phenotypes to be measured and can benefit dramatically from increased SNP density (Ross-Ibarra *et al.* 2007; Walsh 2008). We note that although we identified interesting genomic regions without the use of phenotype data in our two panels, the approach is not “phenotype-free,” but rather the result of repeated strong phenotypic selection.

The fact that we identified signals of selection for FHB resistance that are substantiated by previous mapping efforts in barley suggests that this approach may also be effective when applied to crop species without previous information from QTL mapping. Application of inexpensive genotyping to any breeding population that has a defined history of breeding and selection should provide valuable insight into the genetic architecture of the traits under selection and guidance for marker-based breeding efforts.

## Supplementary Material

Supporting Information
