# Supplementary material for: Comparative Analyses Identify the Contributions of Exotic Donors to Disease Resistance in a Barley Experimental Population
Source: G3 (Bethesda). 2013 Nov 1;3(11):1945–53. doi: 10.1534/g3.113.007294 (PMC3815057; doi:10.1534/g3.113.007294)
Supplement: Supporting Information [file supp_3_11_1945__index.html]

Comparative Analyses Identify the Contributions of Exotic Donors to Disease Resistance in a Barley Experimental Population — Supporting Information 

# Comparative Analyses Identify the Contributions of Exotic Donors to Disease Resistance in a Barley Experimental Population

## Supporting Information for Fang *et al.*, 2013

**Files in this Data Supplement:**

- Supporting Information - Figures S1-S10, File S1, and Tables S1-S4 (PDF, 1 MB)
- Figure S1 - SNP positions and allele frequency comparison of the Closed and Reopened panels on each linkage group. (PDF, 710 KB)
- Figure S2 - Population history, N0, N1 and N2 stand for the Ancestral population, the Closed population and the Reopened population. (PDF, 478 KB)
- Figure S3 - (A) Observed SFS in the Ancestral panel. (B) Simulated SFS in the Ancestral panel using a discovery panel with eight chromosomes and a minor allele count of three. (PDF, 443 KB)
- Figure S4 - *FST* value versus minor allele frequency. (PDF, 486 KB)
- Figure S5 - Prior and posterior density of relative size of the Closed panel from simulations. (PDF, 421 KB)
- Figure S6 - The heatmap of bottleneck. (PDF, 465 KB)
- Figure S7 - Prior and posterior density of migration rate from the Ancestral panel to the Reopened panel. (PDF, 419 KB)
- Figure S8 - IBS and LD plot on linkage group 6H. (PDF, 443 KB)
- Figure S9 - IBS between each of the donor lines and their respective progeny in the Reopened panel on 4H and 6H. (PDF, 450 KB)
- Figure S10 - Percent of adjacent SNPs at varying levels of LD in the Closed and Reopened panel. (PDF, 415 KB)
- File S1 - Supplementary Text (PDF, 397 KB)
- Table S1 - The donor line/lines of each line in the Reopened panel. (PDF, 412 KB)
- Table S2 - The observed pairwise diversity for each linkage group and the median of simulated pairwise diversity in the Ancestral panel, Closed, and Reopened panel. (PDF, 397 KB)
- Table S3 - Markers from previous studies that are within or flanking (~5 cM) the high *FST* blocks and their estimated positions. (PDF, 515 KB)
- Table S4 - BOPA, POPA and SCRI SNPs within genes of known function in the high *FST* blocks and their respective gene products. (PDF, 512 KB)
