# Supplementary material for: Comparative Analyses Identify the Contributions of Exotic Donors to Disease Resistance in a Barley Experimental Population
Source: G3 (Bethesda). 2013 Nov 1;3(11):1945–53. doi: 10.1534/g3.113.007294 (PMC3815057; doi:10.1534/g3.113.007294)
Supplement: Supporting Information [file supp_g3.113.007294_007294SI.pdf]

G3: Genes | Genomes | Genetics

Comparative analyses identify the contributions of exotic donors to disease resistance in a barley experimental population

Zhou Fang[*], Amber Eule-Nashoba[*], Carol Powers[*,1], Thomas Y. Kono[*], Shohei Takuno[§],
Peter L. Morrell[*,2], Kevin P. Smith[*,2]
*Department of Agronomy and Plant Genetics, University of Minnesota, St. Paul, Minnesota 55108
§The Graduate University for Advanced Studies, Hayama, Kanagawa 240-0193, Japan

[1]Present address: Trait Genetics and Technologies, Dow AgroSciences, Indianapolis, Indiana 46268
[2]Corresponding authors: Peter L. Morrell or Kevin P. Smith at the address above or email: pmorrell@umn.edu or smith376@umn.edu

DOI: 10.1534/g3.113.007294

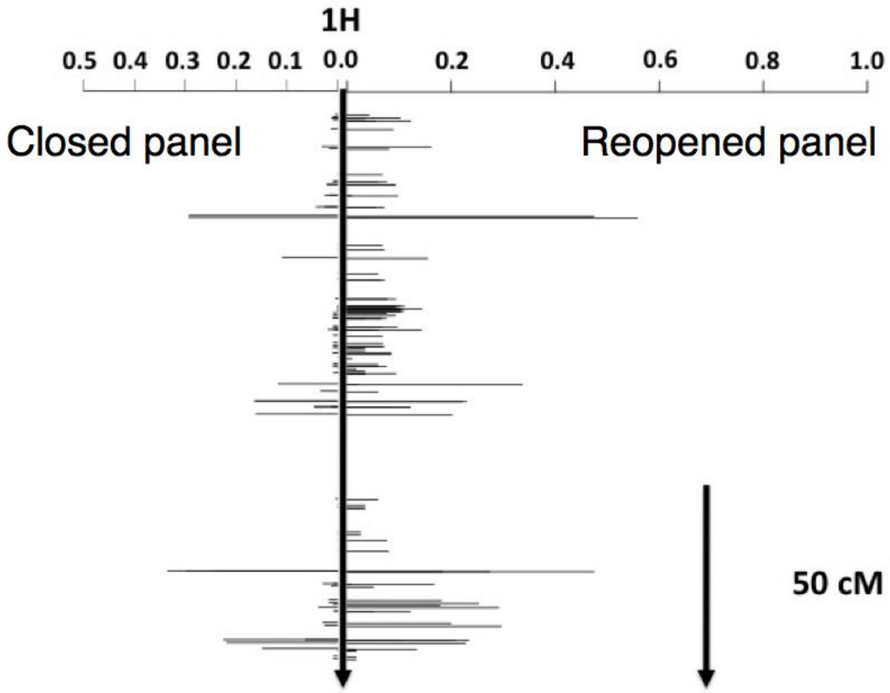

Figure S1 SNP positions and allele frequency comparison of the Closed and Reopened panels on each linkage group. The frequency of the minor allele in the Closed panel is shown. The increase in frequency of some SNPs in the Reopened panel results in SNP states exceeding 50% in frequency. The red lines correspond to the SNPs in the high F_{ST} blocks.

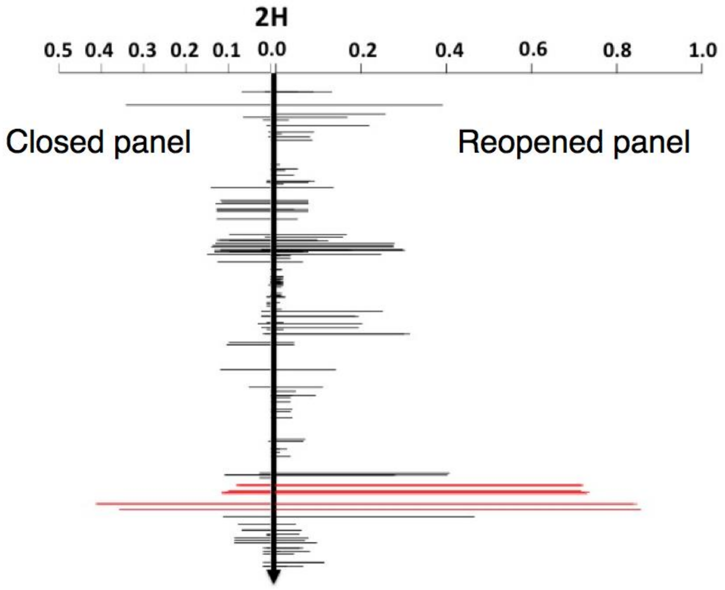

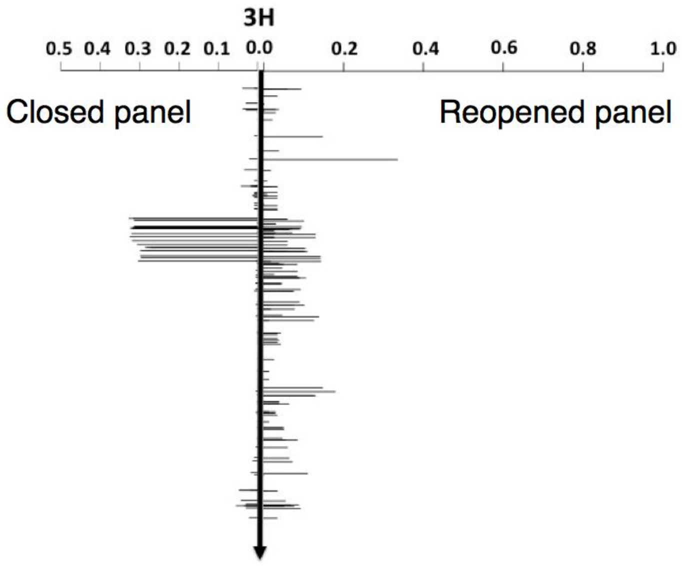

Figure S1 cont.

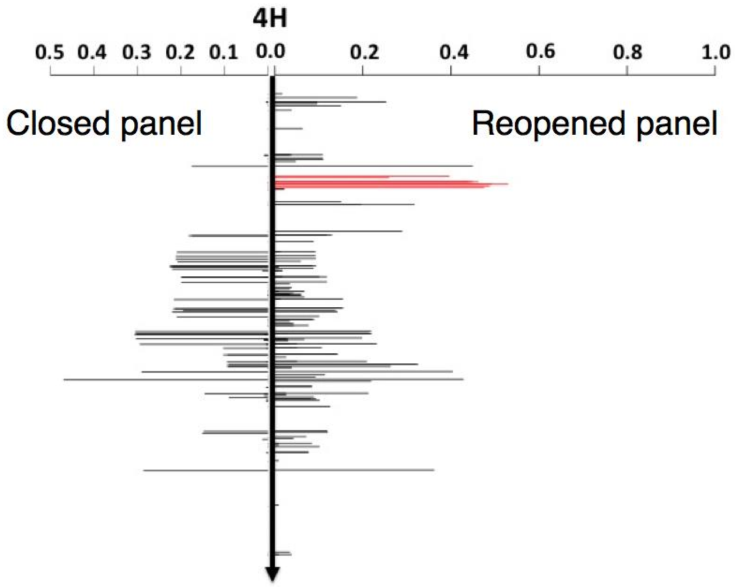

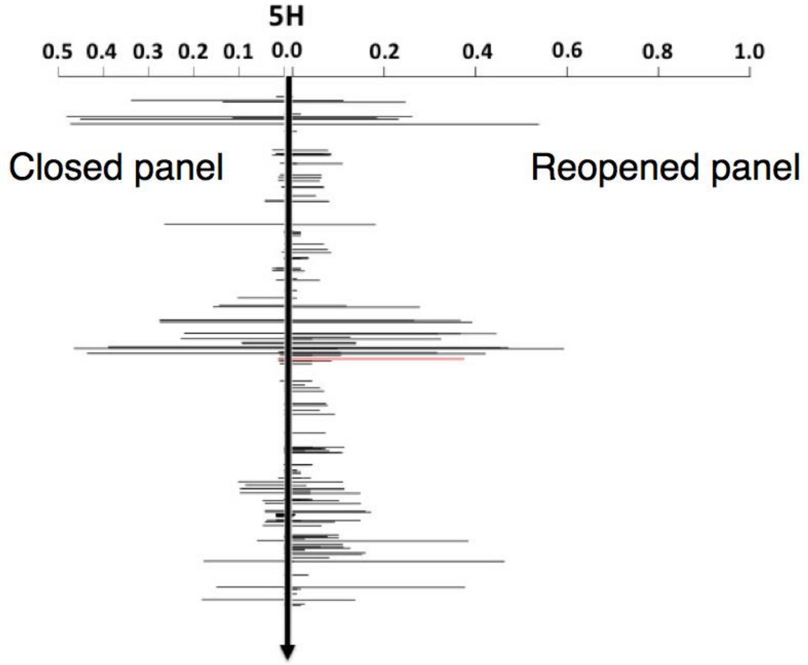

Figure S1 cont.

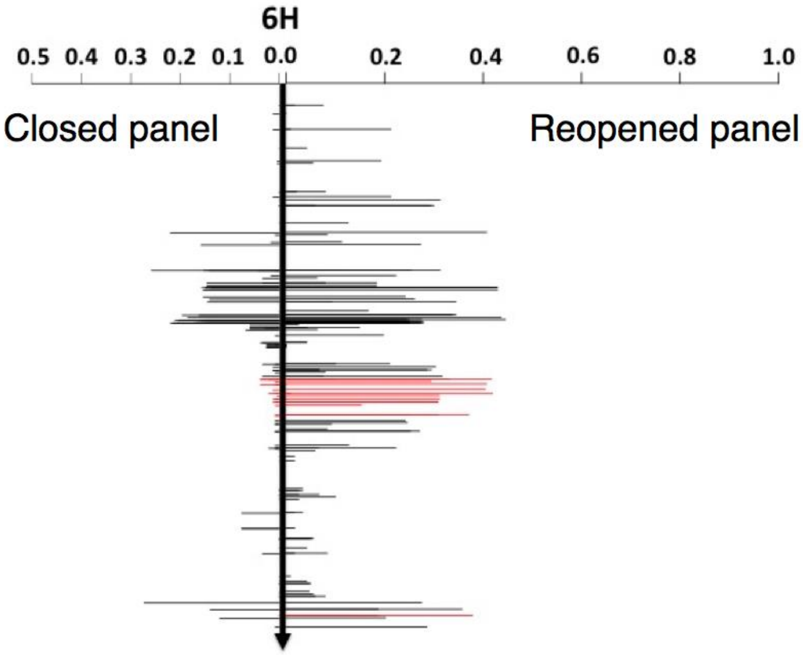

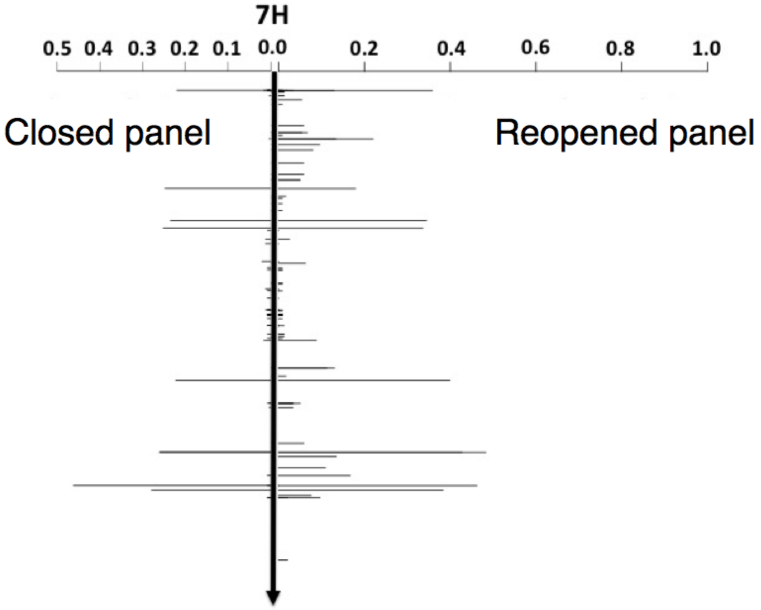

Figure S1 cont.

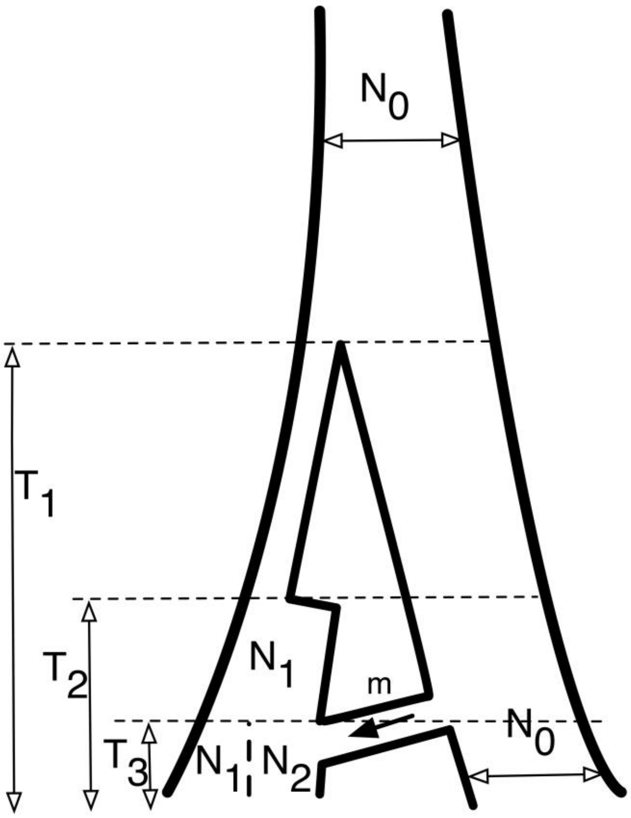

Figure S2 Population history, N_0, N_1 and N_2 stand for the Ancestral population, the Closed population and the Reopened population. T_1 is the start of the bottleneck population, ~8000 generations before present. T_2 is the end of the bottleneck and T_3 is the start of the Reopened population, ~15 generations before present. Migration is from the Ancestral population to the Reopened population.

A

B

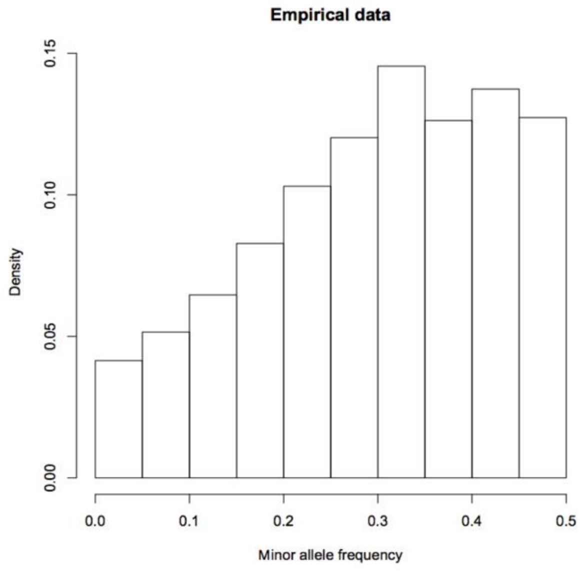

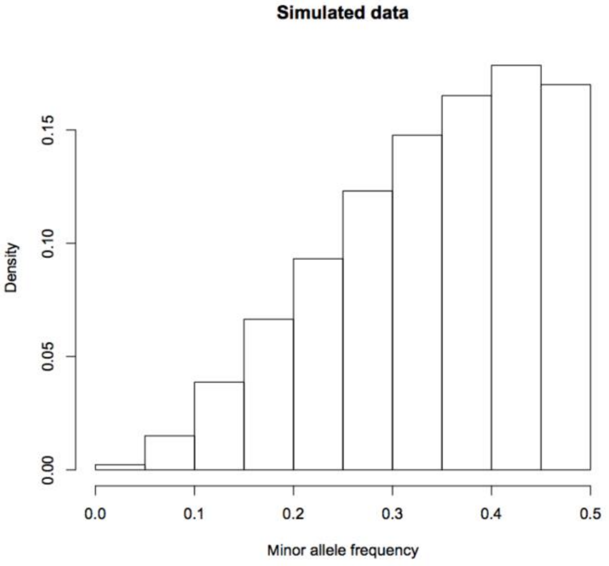

Figure S3 (A) Observed SFS in the Ancestral panel. (B) Simulated SFS in the Ancestral panel using a discovery panel with eight chromosomes and a minor allele count of three.

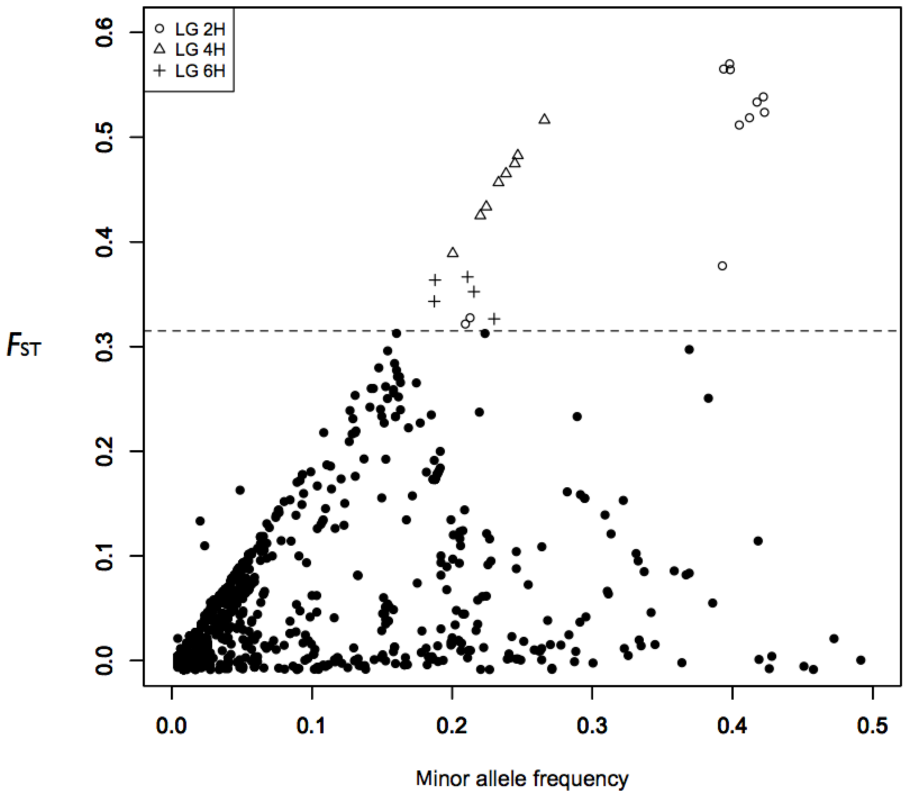

Figure S4 F_{ST} value versus minor allele frequency. The horizontal dashed line corresponds to genome-wide 97.5th percentile of F_{ST} values. All SNPs below the threshold are shown as solid black points. SNPs above the threshold are shown in three symbols corresponding to each of the three linkage groups. Minor allele frequency is based on the whole dataset, including both the Closed and Reopened panels.

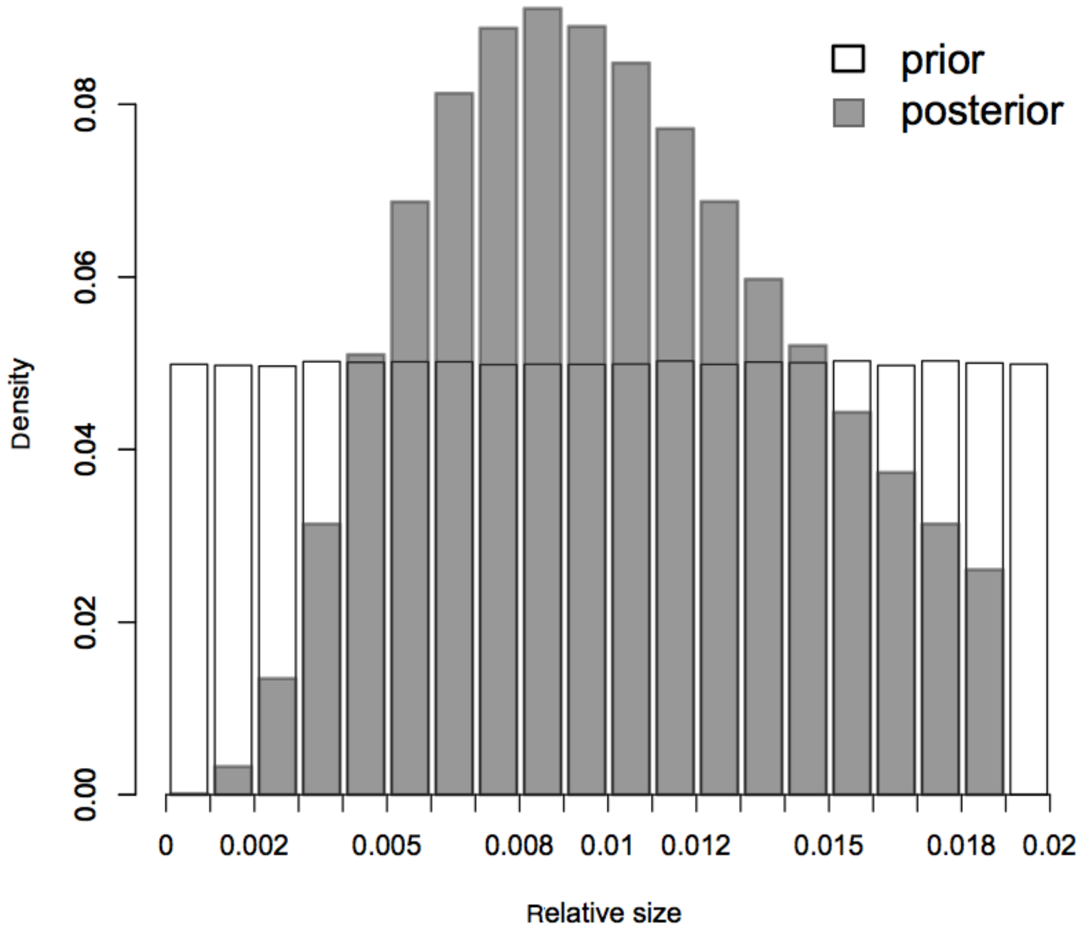

Figure S5 Prior and posterior density of relative size of the Closed panel from simulations.

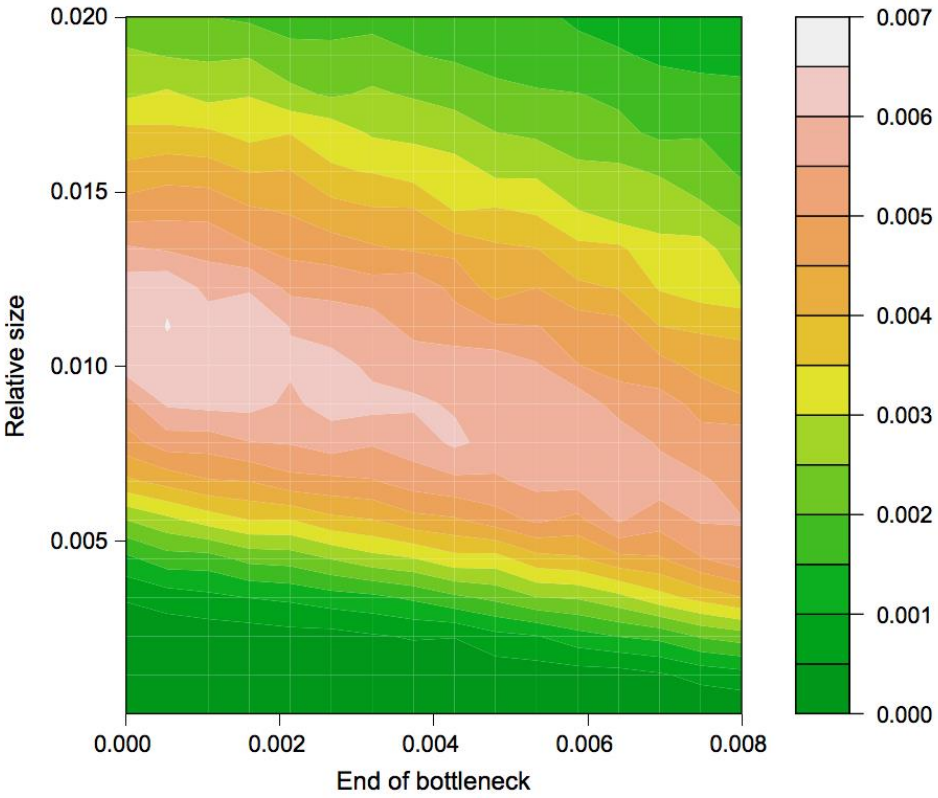

Figure S6 The heatmap of bottleneck. The x-axis is the timing of the end of the bottleneck. The y-axis is the relative size of the bottleneck.

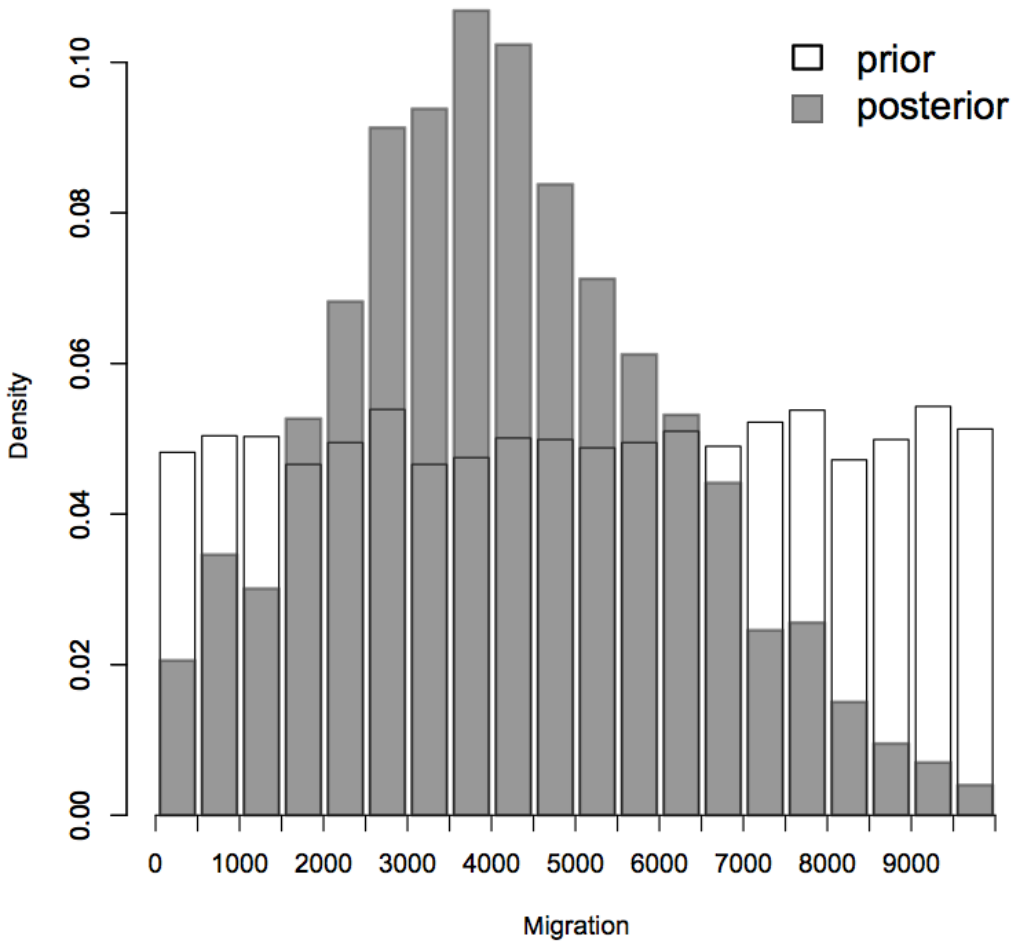

Figure S7 Prior and posterior density of migration rate from the Ancestral panel to the Reopened panel.

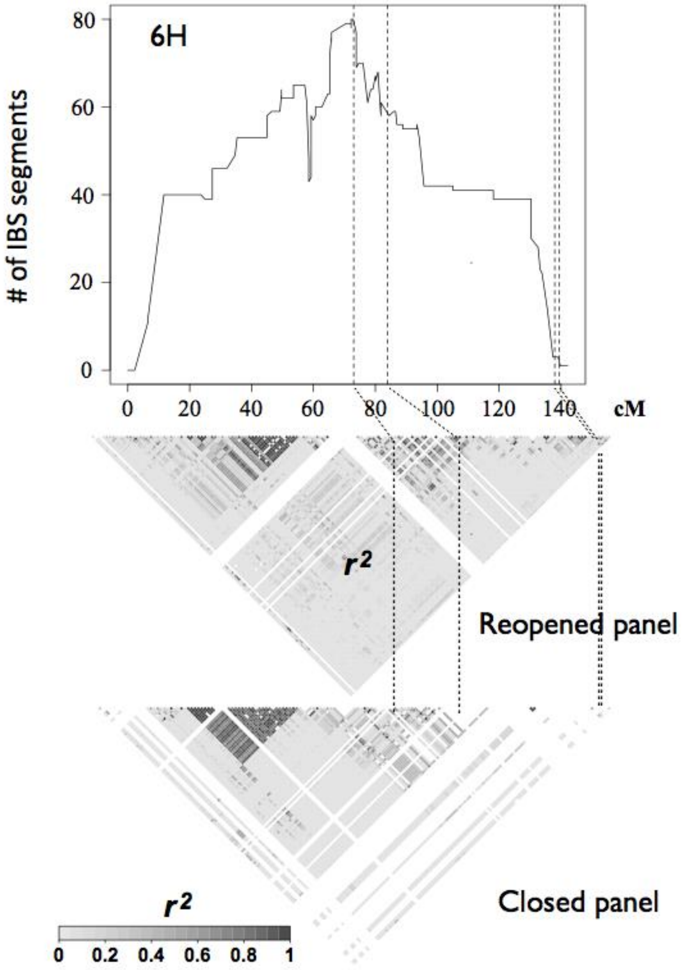

Figure S8 IBS and LD plot on linkage group 6H. The upper panel shows the number of IBS segments between the donor lines and their progeny in the Reopened panel. The vertical dashed lines delimit the high F_{ST} block. The middle and lower panels are the LD heatmaps of the Reopened and Closed panels respectively.

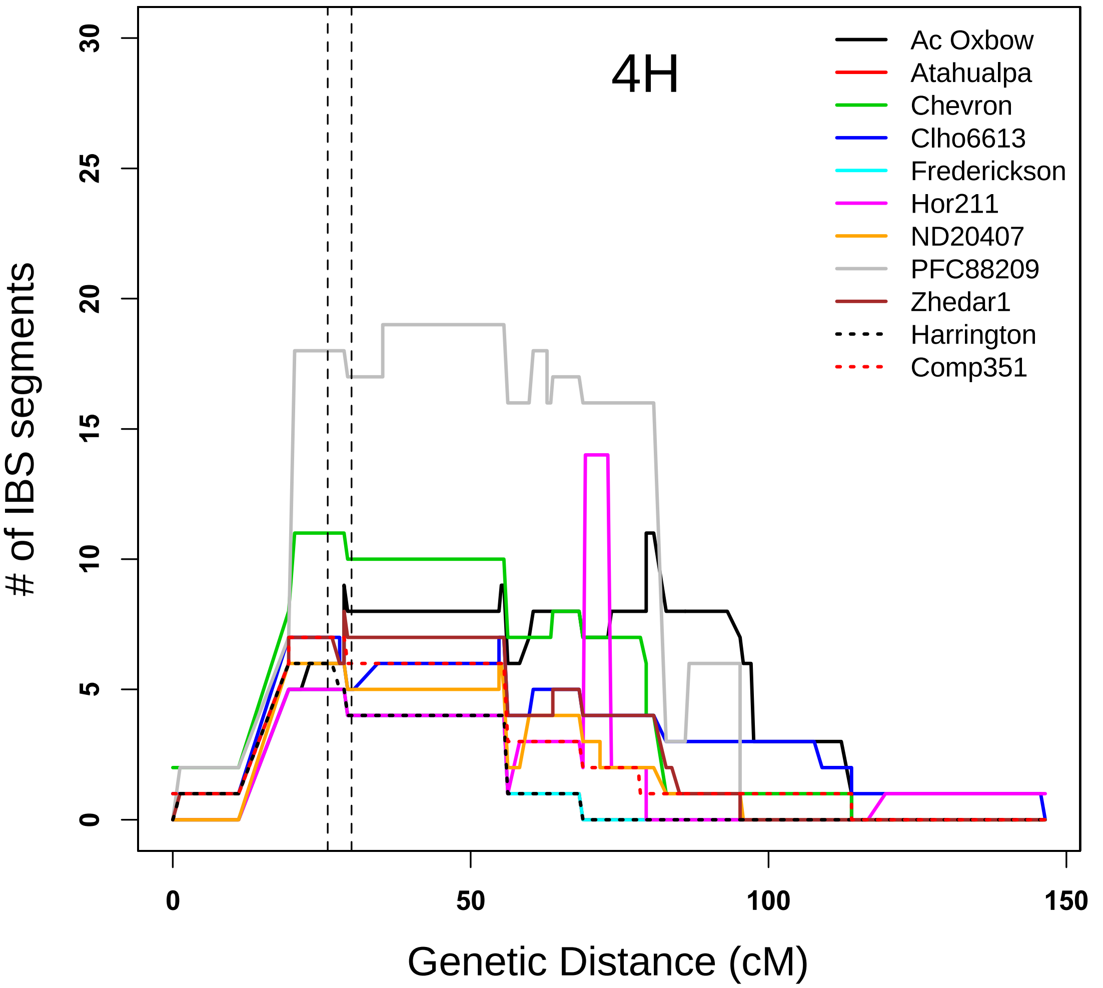
4H
Ac Oxbow
Atahualpa
Chevron
Clho6613
Frederickson
Hor211
ND20407
PFC88209
Zhedar1
Harrington
Comp351
# of IBS segments
0
5
10
15
20
25
30
0
50
100
150
Genetic Distance (cM)

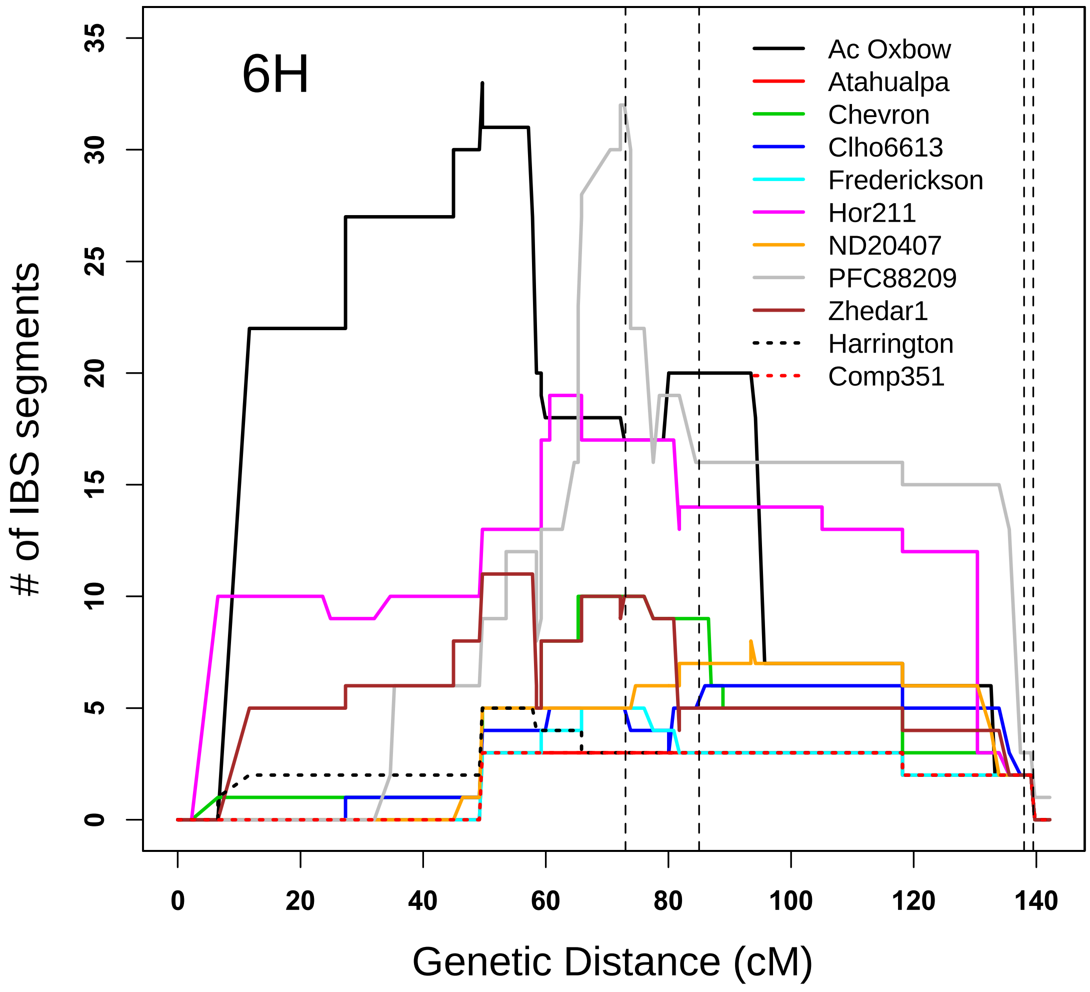

Figure S9 IBS between each of the donor lines and their respective progeny in the Reopened panel on 4H and 6H. The vertical dashed lines delimit the high F_{ST} block.

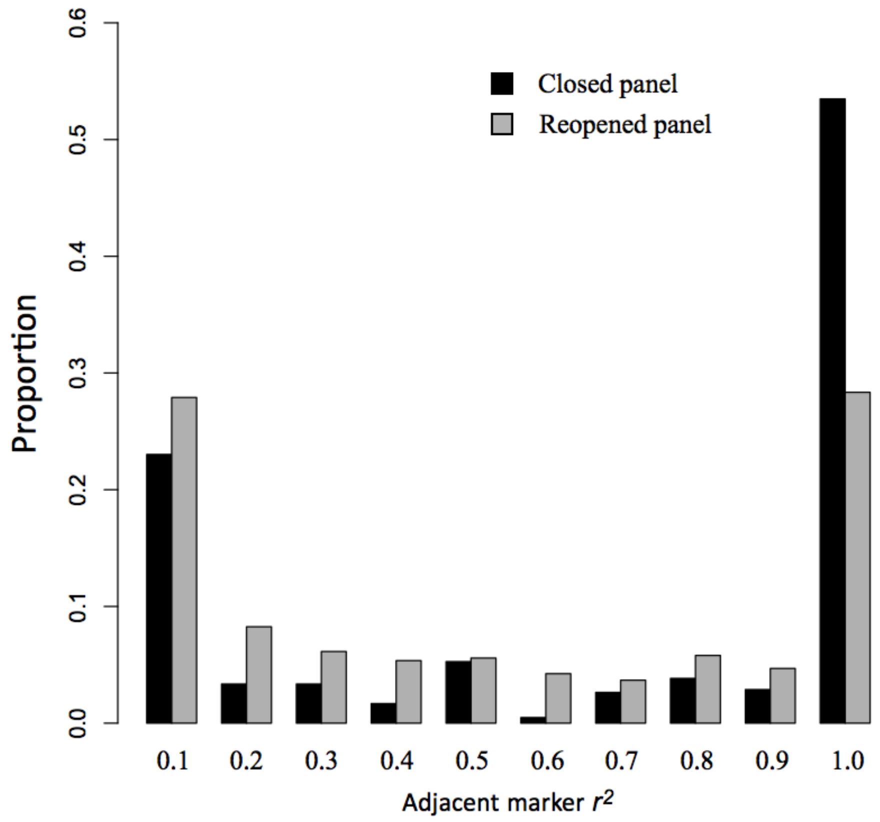

Figure S10 Percent of adjacent SNPs at varying levels of LD in the Closed and Reopened panel.

File S1

Supplementary Text

The Minnesota (MN) barley breeding population (the basis of the Closed panel) has greatly reduced diversity relative to donor lines (Table 1). To generate simulations consistent with this difference in diversity among populations, we simulate the establishment of bottleneck associated with the establishment of the MN population. In ms simulations, we use values U(0.000025, 0.008) and U(0, 0.02) for the end of the bottleneck and relative size of the Closed panel. The population represented by the Reopened panel started ~15 generations ago. For scaling in ms, with time scaled in $4*N_0$, we use N_0 = 150,000 based on $\theta = 4N_0\mu = 0.003$ and $\mu = 5*10^{-9}$, where μ is mutation rate per site per generation. We assume one generation per year. The bottleneck started at $8000/4*N_0 = 0.013$. The Reopened panel started 15 generations before present, $15/4*N_0 = 0.000025$. The end of the bottleneck can be anytime between the start of the Reopened panel (0.000025) and the start of the bottleneck (0.013). Therefore, the relative size of the Closed panel is sampled from a uniform distribution U(0, 0.02). The end of bottleneck is also sampled from a uniform distribution U(0.000025, 0.013). Based on initial simulations, we refined the interval to be U(0.000025, 0.008) in the final simulation.

The command line for simulating the ancestral population and the Closed panel is:
ms 240 1000000 -t 150 -r 1000 1000 -I 2 120 120 -n 2 0.025 -en tbs 2 tbs -ej 0.013 2 1

The command line for simulating all populations is
ms 360 100000 -t 150 -r 1000 1000 -I 3 120 120 120 -n 2 0.025 -en 0.0015 2 0.01 -n 3 0.09 -ej 0.0133 2 1 -m 3 1 tbs -ej 0.000025 3 2 0

Table S1 The donor line/lines of each line in the Reopened panel.

Reopened lines	Donor lines
C113.004	Chevron
C119.002	Chevron
FEG59.09	Ac Oxbow
FEG60.27	BT463
FEG61.37	Clho6613
FEG63.16	Chevron
FEG63.56	Chevron
FEG65.02	Zhedar1
FEG66.05	Zhedar1
FEG66.08	Zhedar1
FEG66.21	Zhedar1
FEG66.31	Zhedar1
FEG67.12	Frederickson
FEG69.24	PFC88209
FEG69.38	PFC88209
FEG73.13	Hor211
FEG73.49	Hor211
FEG74.18	Hor211
FEG74.19	Hor211
FEG75.39	Hor211
FEG80.06	Zhedar1
FEG80.53	Zhedar1
FEG81.58	Harrington
FEG81.60	Harrington
FEG82.16	Chevron
FEG86.03	Hor211
FEG86.53	Hor211
FEG88.73	Atahualpa, Zhedar1
FEG88.87	Atahualpa, Zhedar1
FEG89.73	Hor211
FEG90.31	Zhedar1, Atahualpa
FEG90.35	Zhedar1, Atahualpa
FEG91.28	PFC88209, Frederickson
FEG93.12	Frederickson
FEG93.36	Frederickson
FEG94.20	Zhedar1

FEG94.41	Zhedar1
FEG96.06	Ac Oxbow
FEG96.55	Ac Oxbow
FEG97.14	Ac Oxbow
FEG97.44	Ac Oxbow
FEG98.53	PFC88209
FEG99.10	Ac Oxbow
FEG99.51	Ac Oxbow
FEG100.17	Zhedar1
FEG100.33	Zhedar1
FEG100.41	Zhedar1
FEG100.47	Zhedar1
FEG103.44	Ac Oxbow, Harrington
FEG103.45	Ac Oxbow, Harrington
FEG104.63	Zhedar1
FEG104.89	Zhedar1
FEG105.33	PFC88209
FEG105.59	PFC88209
FEG109.13	Ac Oxbow
FEG109.44	Ac Oxbow
FEG109.54	Ac Oxbow
FEG111.10	Ac Oxbow, Zhedar1
FEG111.13	Ac Oxbow, Zhedar1
FEG111.24	Ac Oxbow, Zhedar1
FEG112.14	Ac Oxbow, Atahualpa
FEG113.85	Ac Oxbow, Zhedar1
FEG114.33	Clho6613
FEG116.05	Zhedar1
FEG117.24	Zhedar1
FEG118.05	PFC88209
FEG118.41	PFC88209
FEG118.69	PFC88209
FEG121.03	Zhedar1, Ac Oxbow
FEG121.16	Zhedar1, Ac Oxbow
FEG121.29	Zhedar1, Ac Oxbow
FEG121.43	Zhedar1, Ac Oxbow
FEG122.36	Hor211, PFC88209
FEG122.50	Hor211, PFC88209
FEG122.92	Hor211, PFC88209

FEG124.35	PFC88209
FEG125.46	Zhedar1
FEG125.69	Zhedar1
FEG126.08	Zhedar1
FEG126.14	Zhedar1
FEG129.41	Frederickson
FEG129.60	Frederickson
FEG132.05	Zhedar1, Frederickson
FEG132.63	Zhedar1, Frederickson
FEG138.08	Zhedar1, Hor211
FEG138.27	Zhedar1, Hor211
FEG141.18	Ac Oxbow
FEG141.20	Ac Oxbow
FEG142.13	Zhedar1, Hor211
FEG142.28	Zhedar1, Hor211
FEG142.55	Zhedar1, Hor211
FEG144.21	Ac Oxbow, Hor211
FEG144.27	Ac Oxbow, Hor211
FEG144.68	Ac Oxbow, Hor211
FEG146.09	Frederickson
FEG146.46	Frederickson
FEG146.68	Frederickson
FEG147.03	Zhedar1, Atahualpa
FEG147.14	Zhedar1, Atahualpa
FEG147.63	Zhedar1, Atahualpa
FEG148.22	Ac Oxbow
FEG148.56	Ac Oxbow
FEG149.18	ND20407
FEG149.65	ND20407
FEG150.42	ND20493
FEG150.49	ND20493
FEG153.22	Zhedar1
FEG155.07	Ac Oxbow
FEG156.09	Zhedar1
FEG161.03	Ac Oxbow
FEG162.22	Ac Oxbow
FEG163.21	Zhedar1
FEG164.33	Hor211, PFC88209
FEG166.38	Zhedar1

FEG168.09	Comp351
FEG169.47	Hor211
FEG170.07	Hor211
FEG172.40	Hor211
FEG175.57	Zhedar1, Hor211

Table S2 The observed pairwise diversity (scaled by the number of segregating sites) for each linkage group and the median of simulated pairwise diversity in the Ancestral panel, Closed, and Reopened panel.

LG	Ancestral panel	Closed panel	Reopened panel
1H	0.049	0.002	0.004
2H	0.060	0.004	0.004
3H	0.072	0.001	0.005
4H	0.057	0.001	0.005
5H	0.068	0.004	0.007
6H	0.059	0.007	0.011
7H	0.044	0.002	0.002
Simulated	0.054	0.007	0.014

Table S3 Markers from previous studies that are within or flanking (~5 cM) the high F_{ST} blocks and their estimated positions.

Linkage group	Marker	Position (cM)
2H	ABC252	141.89 – 142.42
	CDO373	
	F3hA	
	MWG5208	
	pKABA1	
	Ebmc0415	142.42 – 143.72
	Cnx1	
	BCD135	146.05 – 147.93
	Gln2	
	KG004.1	
	KG004.2	
	ABC157	148.58 – 150.55
	Zeo1	150.55 – 152.64
4H	HVM40	19.27 – 20.26
	CDO669A	20.68 – 21.33
	Ole1	24.49 – 25.23
	BCD402B	26.47
	CDO542	29.08 – 29.49
	DsT-29	
	CDO122	30.10
	BCD351D	30.10 – 31.41
	INT-C	30.37
	MWG635A	31.41 – 32.62
	BCD265B	34.92 – 37.54
	BCD808B	
5H	Scssr05939	94.25 – 94.66
	ksuD17	65.89 – 69.87
	G57	
	ABC163	71.52 – 72.17
	ABG379	
	Bmac0218C	73.18 – 73.98
	ABG388	74.60 – 75.34
	CDO507	
	ABC175	77.82 – 78.46
	RZ323	

6H	ksuA3D	79.34 – 80.13
	ABC1708	
	Scsnp21226	81.05 – 82.53
	MWG820	83.71 – 85.17
	cMWG684D	88.08 – 88.78
	MWG514	136.17 – 137.84
	MWG798A	
	ABG725	138.49 – 140.92
	DAK213C	
	DsT-71	

Table S4 BOPA, POPA and SCRI SNPs within genes of known function in the high F_{ST} blocks and their respective gene products.

LG	SNP	GenBankID	cM	Silent	Gene	Product
2H	SCRI_RS_173017	NM_001073041	139.9	No	Os12g0256900	hypothetic protein
	11_10446	XM_003560174	140.69	No	LOC100837523	serine carboxypeptidase-like
	11_20480	AY162186	140.69	Yes	exin1	Extracellular invertase
	SCRI_RS_15119	DQ163025	141.5	Yes	VTE5	phytol kinase
	11_21459	AM039897	143.18	No	ahh1	S-adenosyl-L-homocysteine hydrolase
	2_1484	AB058924	143.71	Yes	HvPKABA1	protein kinase HvPKABA1
	11_10656	XM_003580700	145.69	Yes	LOC100826196	U3 small nucleolar ribonucleoprotein protein IMP3-like
	11_10383	AY136627	147.37	Yes	Ha1	plasma membrane P-type proton pump ATPase
	12_30942	GQ169685	147.37	Yes	GS2	plastid glutamine synthetase 2
4H	11_20422	XM_003560743	28	Yes	LOC100820964	microsomal glutathione S-transferase 3-like isoform 1
	SCRI_RS_157832	XM_003560569	30	Yes	LOC100840876	vam6/Vps39-like protein-like
6H	SCRI_RS_143317	XM_003570599	72.9	No	LOC100831957	RINT1-like protein-like
	SCRI_RS_206976	XM_003570630	74.6	No	LOC100841641	microtubule-associated protein TORTIFOLIA1-like
