# Supplementary material for: Comparative Analyses Identify the Contributions of Exotic Donors to Disease Resistance in a Barley Experimental Population
Source: G3 (Bethesda). 2013 Nov 1;3(11):1945–53. doi: 10.1534/g3.113.007294 (PMC3815057; doi:10.1534/g3.113.007294)
Supplement: Supporting Information [file supp_g3.113.007294_FigureS1.pdf]

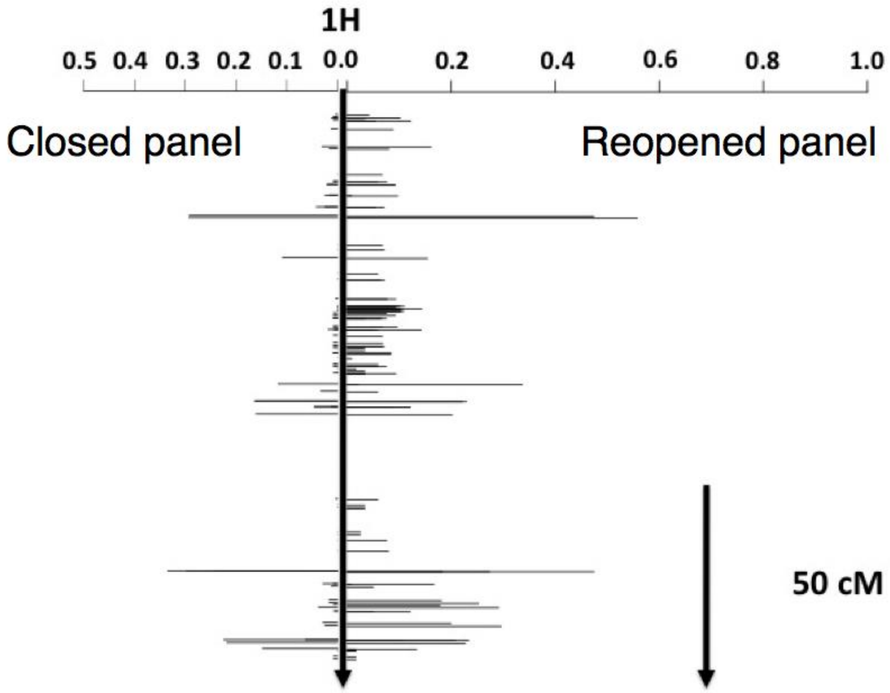

Figure S1 SNP positions and allele frequency comparison of the Closed and Reopened panels on each linkage group. The frequency of the minor allele in the Closed panel is shown. The increase in frequency of some SNPs in the Reopened panel results in SNP states exceeding 50% in frequency. The red lines correspond to the SNPs in the high F_{ST} blocks.

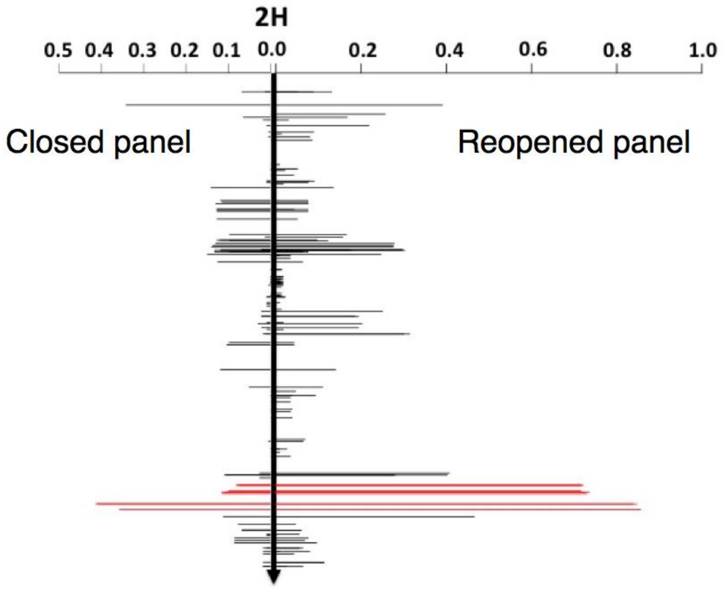

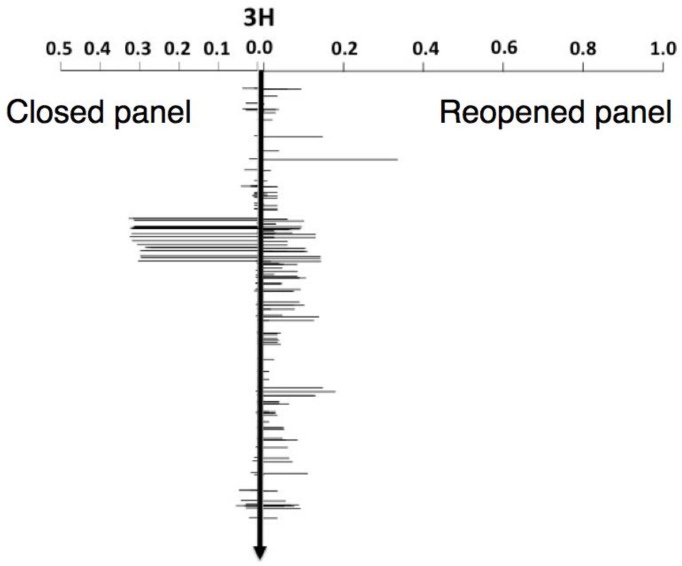

Figure S1 cont.

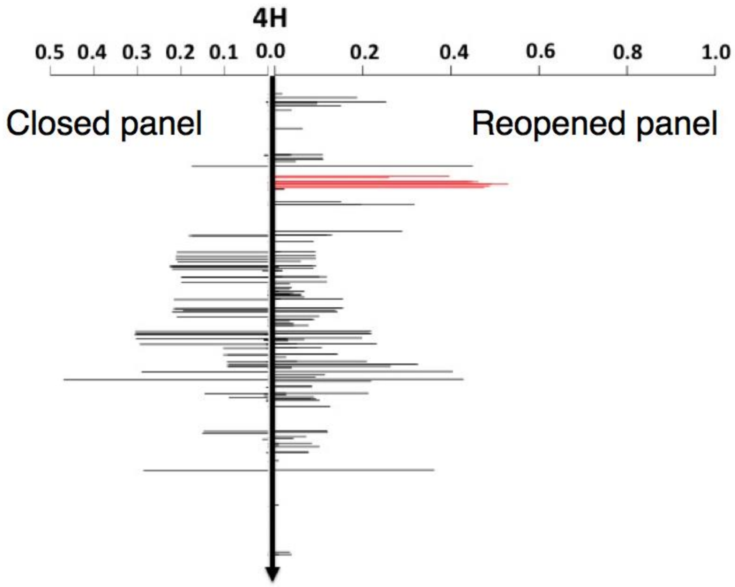

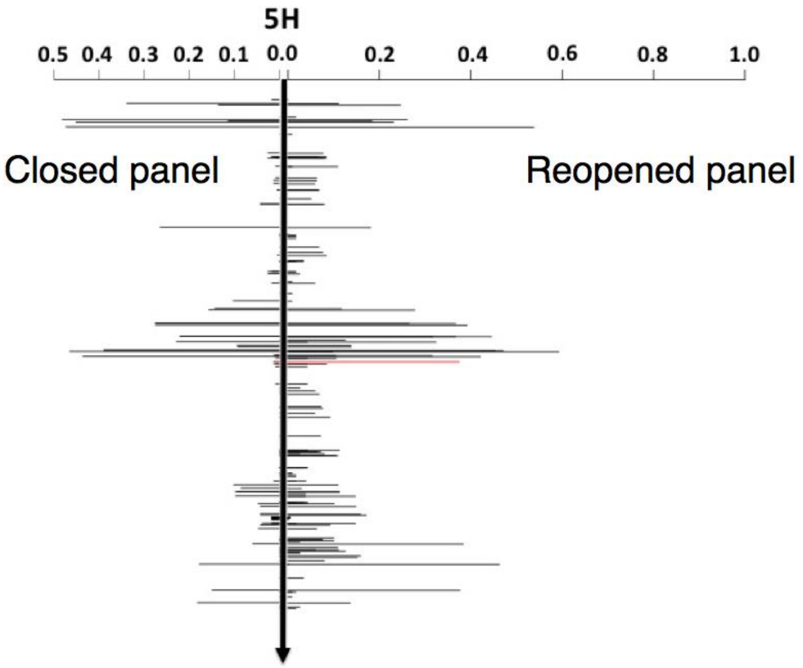

Figure S1 cont.

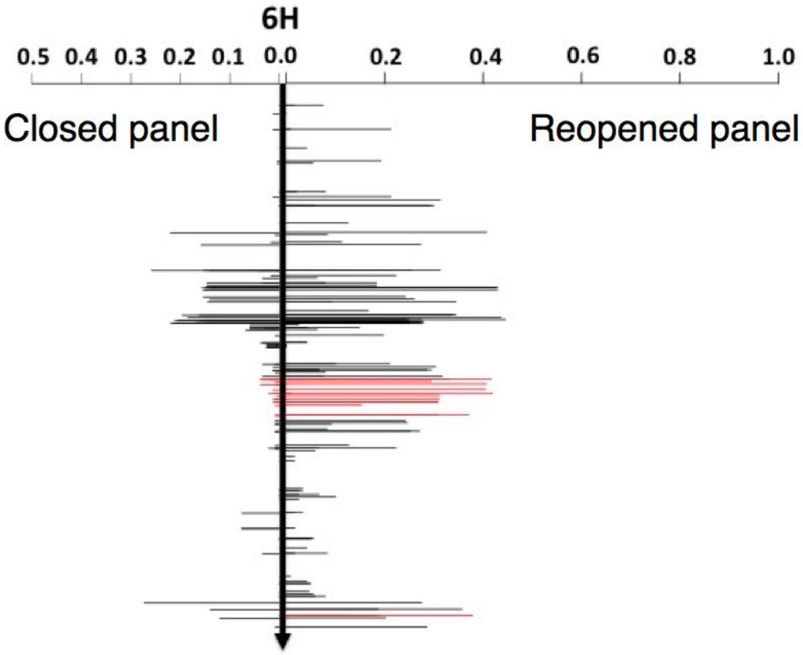

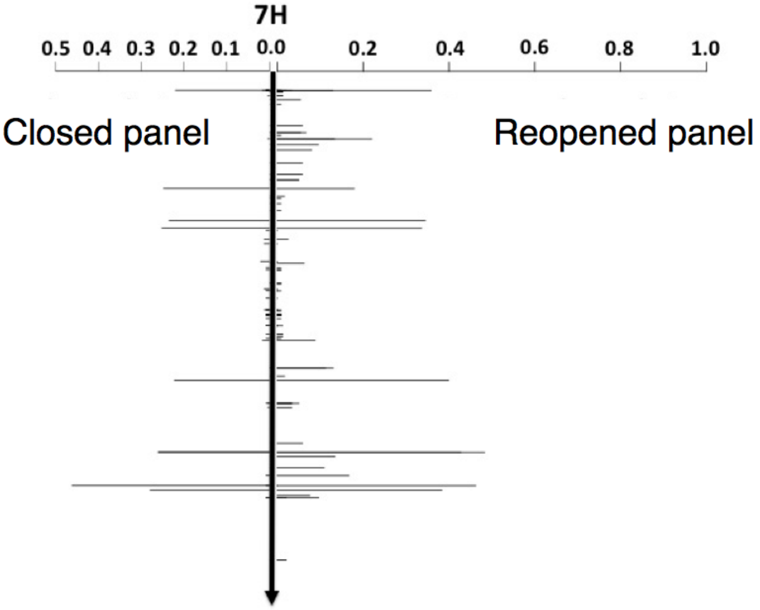

Figure S1 cont.
