# Supplementary material for: Comparative Analyses Identify the Contributions of Exotic Donors to Disease Resistance in a Barley Experimental Population
Source: G3 (Bethesda). 2013 Nov 1;3(11):1945–53. doi: 10.1534/g3.113.007294 (PMC3815057; doi:10.1534/g3.113.007294)
Supplement: Supporting Information [file supp_g3.113.007294_FigureS2.pdf]

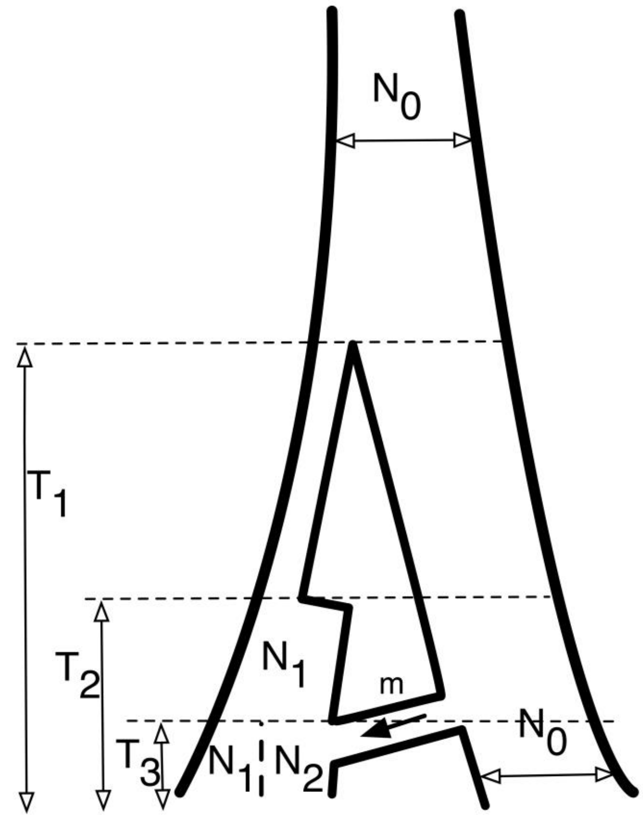

Figure S2 Population history, N_0, N_1 and N_2 stand for the Ancestral population, the Closed population and the Reopened population. T_1 is the start of the bottleneck population, ~8000 generations before present. T_2 is the end of the bottleneck and T_3 is the start of the Reopened population, ~15 generations before present. Migration is from the Ancestral population to the Reopened population.
