# Supplementary material for: Comparative Analyses Identify the Contributions of Exotic Donors to Disease Resistance in a Barley Experimental Population
Source: G3 (Bethesda). 2013 Nov 1;3(11):1945–53. doi: 10.1534/g3.113.007294 (PMC3815057; doi:10.1534/g3.113.007294)
Supplement: Supporting Information [file supp_g3.113.007294_FigureS3.pdf]

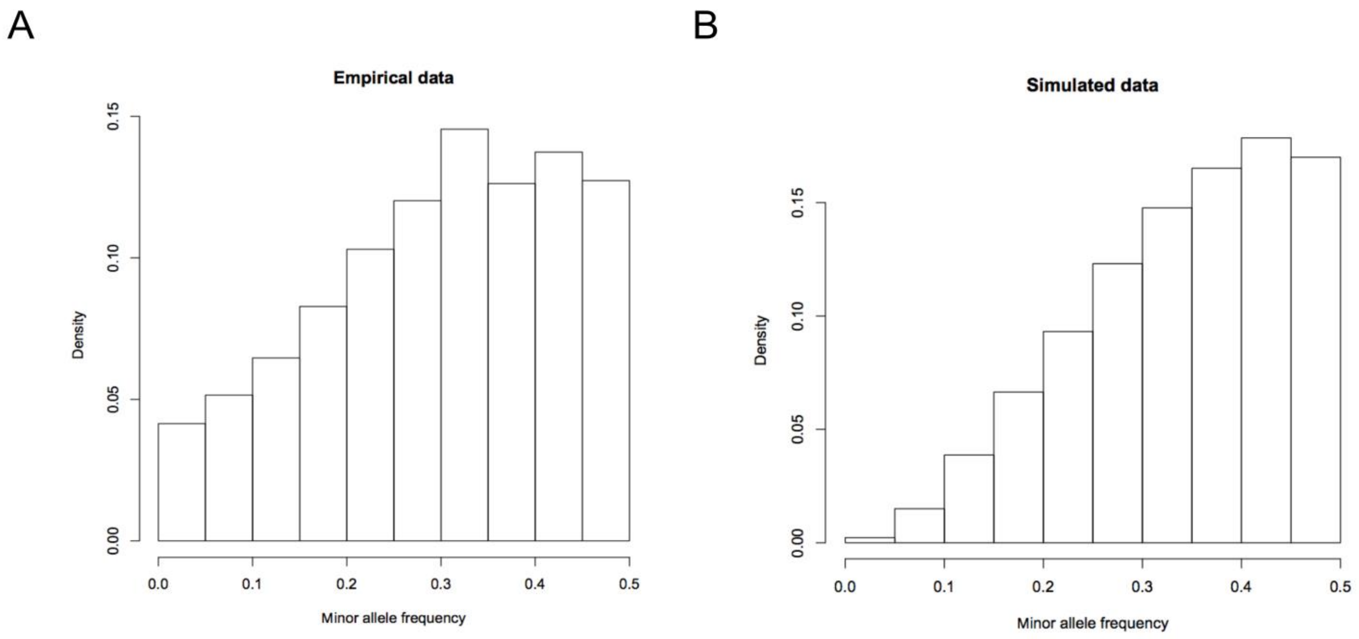

Figure S3 (A) Observed SFS in the Ancestral panel. (B) Simulated SFS in the Ancestral panel using a discovery panel with eight chromosomes and a minor allele count of three.
