# Supplementary material for: Comparative Analyses Identify the Contributions of Exotic Donors to Disease Resistance in a Barley Experimental Population
Source: G3 (Bethesda). 2013 Nov 1;3(11):1945–53. doi: 10.1534/g3.113.007294 (PMC3815057; doi:10.1534/g3.113.007294)
Supplement: Supporting Information [file supp_g3.113.007294_FigureS4.pdf]

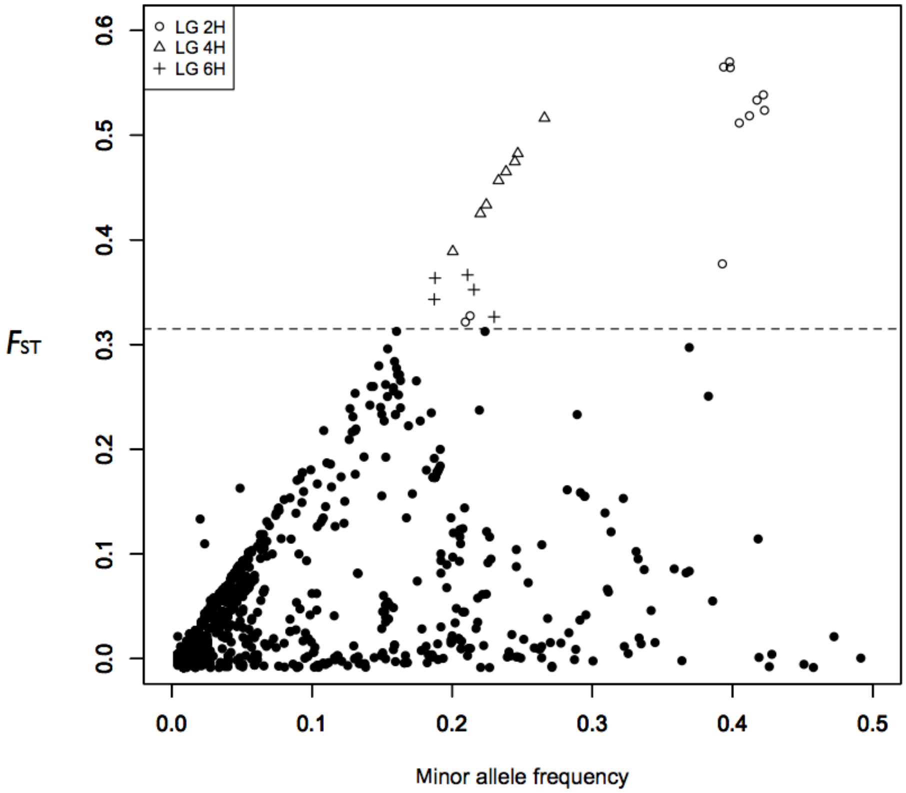

Figure S4 F_{ST} value versus minor allele frequency. The horizontal dashed line corresponds to genome-wide 97.5th percentile of F_{ST} values. All SNPs below the threshold are shown as solid black points. SNPs above the threshold are shown in three symbols corresponding to each of the three linkage groups. Minor allele frequency is based on the whole dataset, including both the Closed and Reopened panels.
