# Supplementary figures and images for: Comparative Analyses Identify the Contributions of Exotic Donors to Disease Resistance in a Barley Experimental Population
Source: G3 (Bethesda). 2013 Nov 1;3(11):1945–53. doi: 10.1534/g3.113.007294 (PMC3815057; doi:10.1534/g3.113.007294)

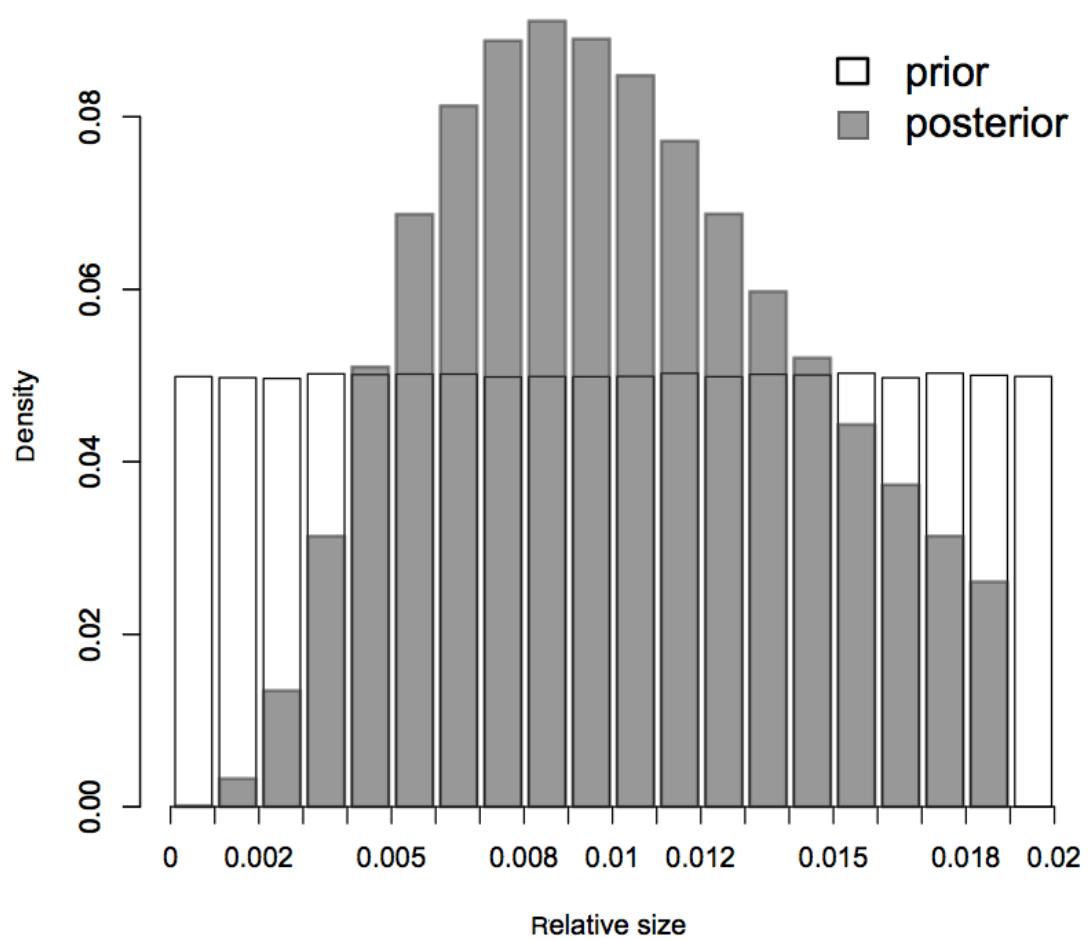

**Figure S5** Prior and posterior density of relative size of the Closed panel from simulations.

Supplement: Supporting Information [file supp_g3.113.007294_FigureS5.pdf]

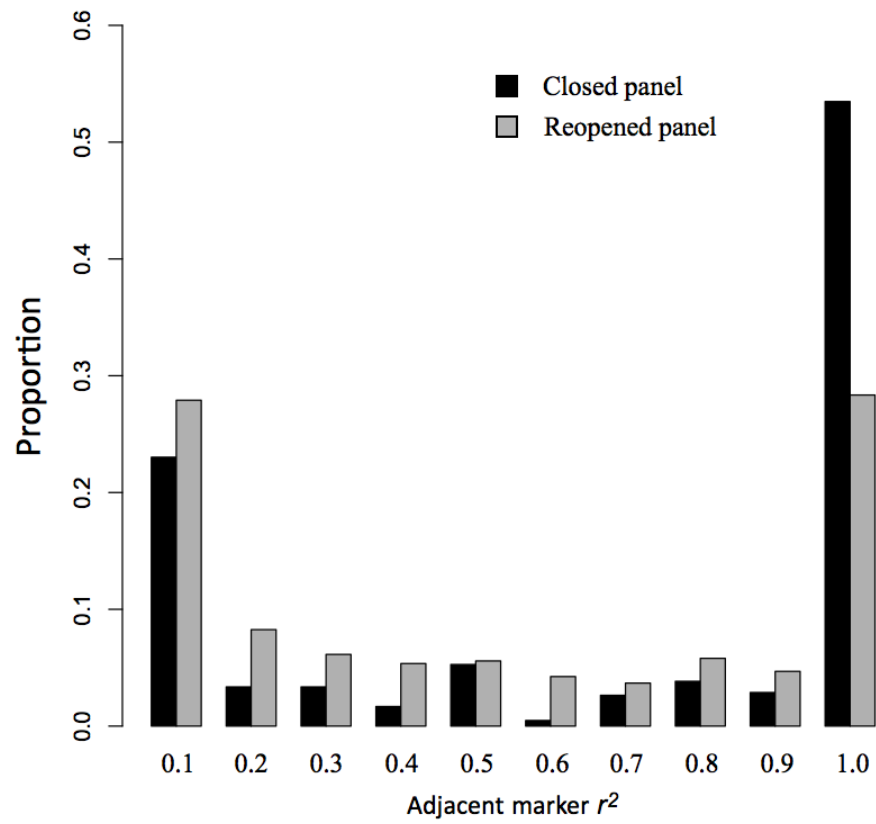

**Figure S10** Percent of adjacent SNPs at varying levels of LD in the Closed and Reopened panel.

Supplement: Supporting Information [file supp_g3.113.007294_FigureS10.pdf]
