# Supplementary material for: Comparative Analyses Identify the Contributions of Exotic Donors to Disease Resistance in a Barley Experimental Population
Source: G3 (Bethesda). 2013 Nov 1;3(11):1945–53. doi: 10.1534/g3.113.007294 (PMC3815057; doi:10.1534/g3.113.007294)
Supplement: Supporting Information [file supp_g3.113.007294_FigureS6.pdf]

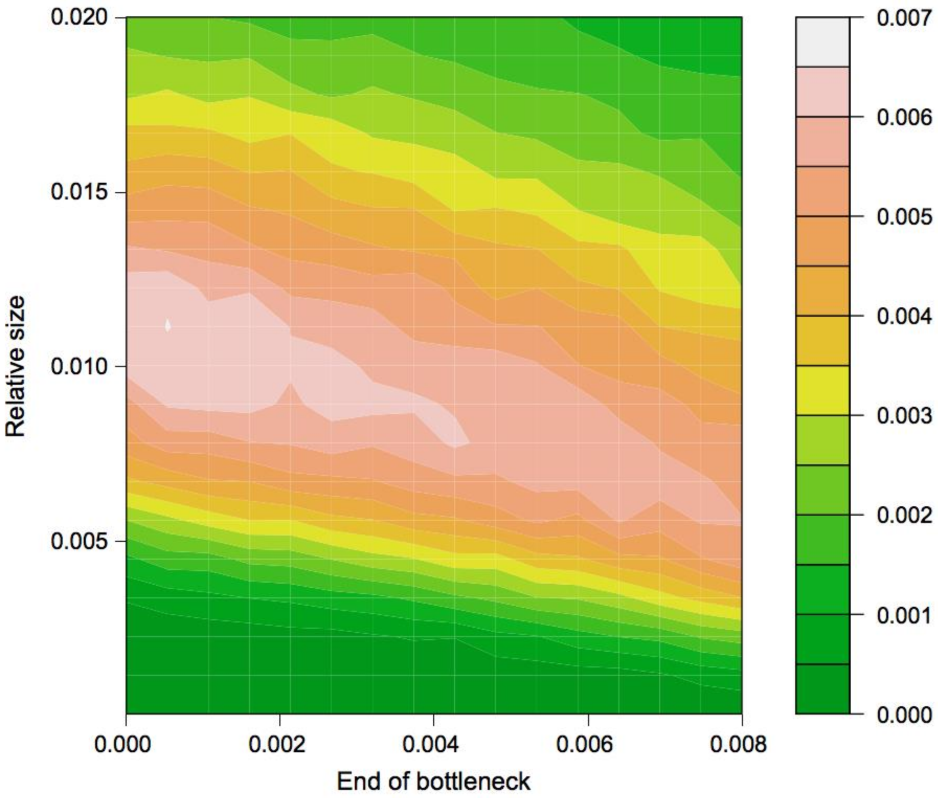

Figure S6 The heatmap of bottleneck. The x-axis is the timing of the end of the bottleneck. The y-axis is the relative size of the bottleneck.
