# Supplementary material for: Comparative Analyses Identify the Contributions of Exotic Donors to Disease Resistance in a Barley Experimental Population
Source: G3 (Bethesda). 2013 Nov 1;3(11):1945–53. doi: 10.1534/g3.113.007294 (PMC3815057; doi:10.1534/g3.113.007294)
Supplement: Supporting Information [file supp_g3.113.007294_FigureS8.pdf]

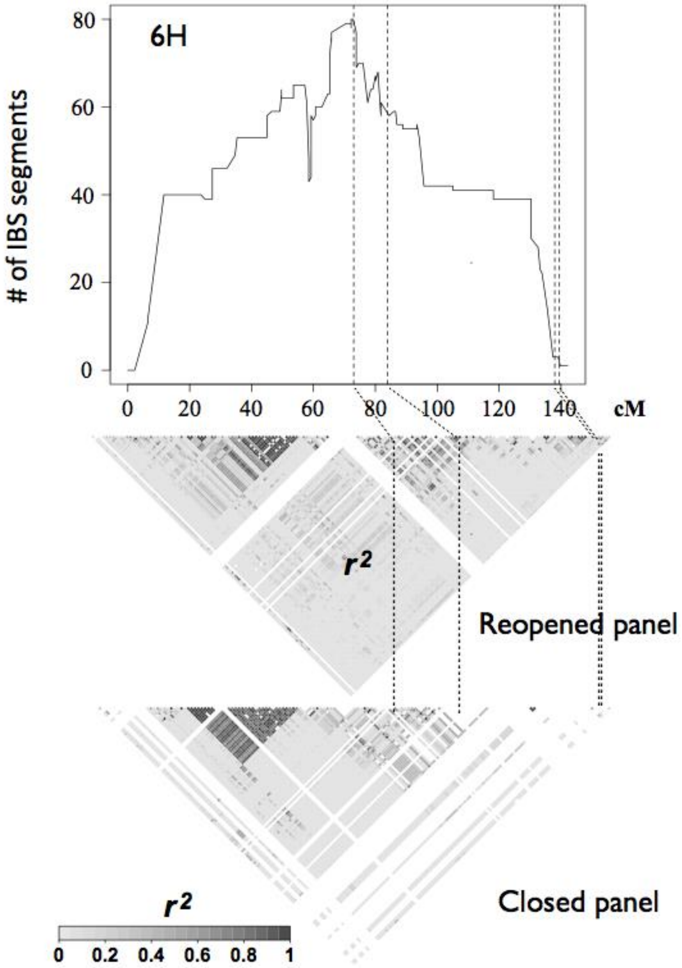

Figure S8 IBS and LD plot on linkage group 6H. The upper panel shows the number of IBS segments between the donor lines and their progeny in the Reopened panel. The vertical dashed lines delimit the high F_{ST} block. The middle and lower panels are the LD heatmaps of the Reopened and Closed panels respectively.
