# Supplementary material for: Comparative Analyses Identify the Contributions of Exotic Donors to Disease Resistance in a Barley Experimental Population
Source: G3 (Bethesda). 2013 Nov 1;3(11):1945–53. doi: 10.1534/g3.113.007294 (PMC3815057; doi:10.1534/g3.113.007294)
Supplement: Supporting Information [file supp_g3.113.007294_FigureS9.pdf]

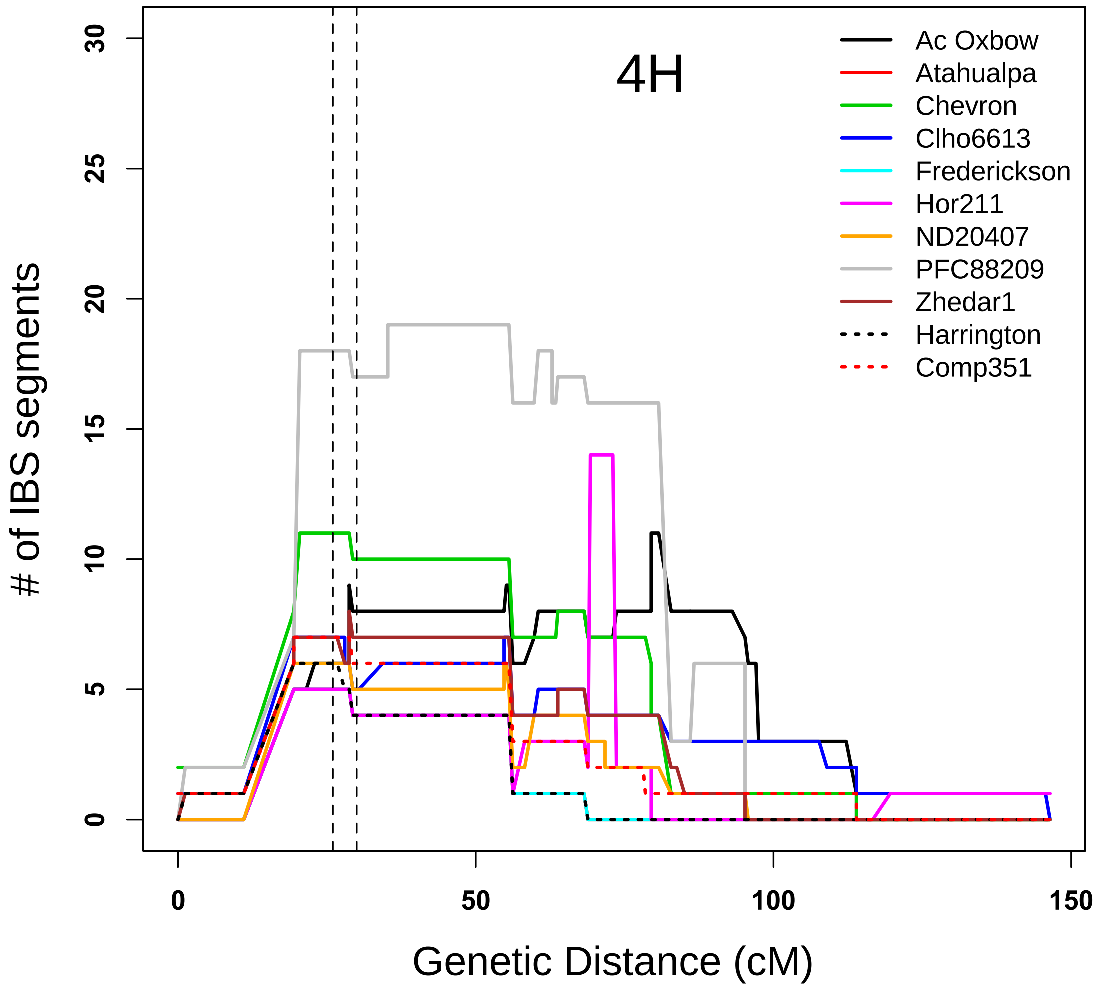
4H
Ac Oxbow
Atahualpa
Chevron
Clho6613
Frederickson
Hor211
ND20407
PFC88209
Zhedar1
Harrington
Comp351
# of IBS segments
0
5
10
15
20
25
30
0
50
100
150
Genetic Distance (cM)

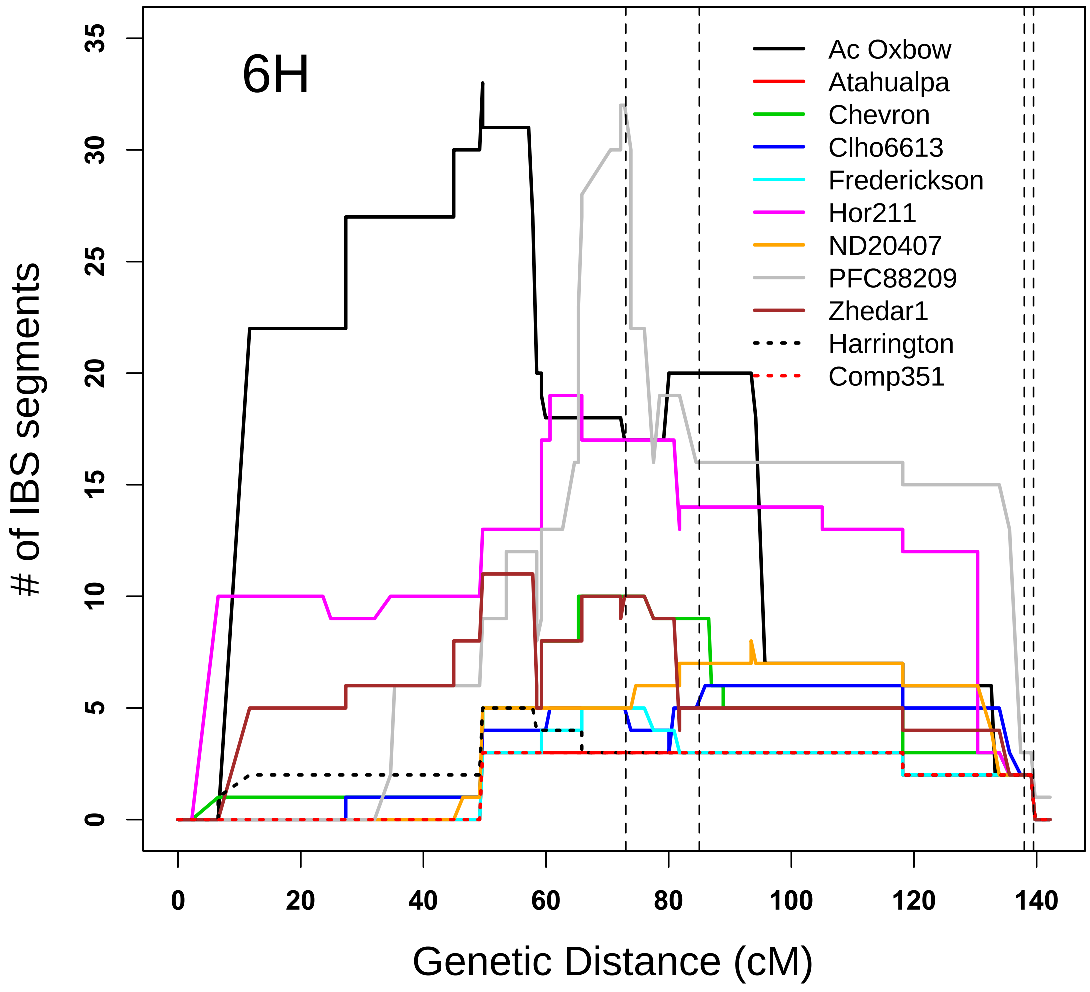

Figure S9 IBS between each of the donor lines and their respective progeny in the Reopened panel on 4H and 6H. The vertical dashed lines delimit the high F_{ST} block.
