# Supplementary material for: Comparative Analyses Identify the Contributions of Exotic Donors to Disease Resistance in a Barley Experimental Population
Source: G3 (Bethesda). 2013 Nov 1;3(11):1945–53. doi: 10.1534/g3.113.007294 (PMC3815057; doi:10.1534/g3.113.007294)
Supplement: Supporting Information [file supp_g3.113.007294_FileS1.pdf]

File S1

Supplementary Text

The Minnesota (MN) barley breeding population (the basis of the Closed panel) has greatly reduced diversity relative to donor lines (Table 1). To generate simulations consistent with this difference in diversity among populations, we simulate the establishment of bottleneck associated with the establishment of the MN population. In ms simulations, we use values U(0.000025, 0.008) and U(0, 0.02) for the end of the bottleneck and relative size of the Closed panel. The population represented by the Reopened panel started ~15 generations ago. For scaling in ms, with time scaled in $4*N_0$, we use $N_0 = 150{,}000$ based on $\theta = 4N_0\mu = 0.003$ and $\mu = 5*10^{-9}$, where μ is mutation rate per site per generation. We assume one generation per year. The bottleneck started at $8000/4*N_0 = 0.013$. The Reopened panel started 15 generations before present, $15/4*N_0 = 0.000025$. The end of the bottleneck can be anytime between the start of the Reopened panel (0.000025) and the start of the bottleneck (0.013). Therefore, the relative size of the Closed panel is sampled from a uniform distribution U(0, 0.02). The end of bottleneck is also sampled from a uniform distribution U(0.000025, 0.013). Based on initial simulations, we refined the interval to be U(0.000025, 0.008) in the final simulation.

The command line for simulating the ancestral population and the Closed panel is:
ms 240 1000000 -t 150 -r 1000 1000 -I 2 120 120 -n 2 0.025 -en tbs 2 tbs -ej 0.013 2 1

The command line for simulating all populations is
ms 360 100000 -t 150 -r 1000 1000 -I 3 120 120 120 -n 2 0.025 -en 0.0015 2 0.01 -n 3 0.09 -ej 0.0133 2 1 -m 3 1 tbs -ej 0.000025 3 2 0
