# Supplementary material for: Comparative Analyses Identify the Contributions of Exotic Donors to Disease Resistance in a Barley Experimental Population
Source: G3 (Bethesda). 2013 Nov 1;3(11):1945–53. doi: 10.1534/g3.113.007294 (PMC3815057; doi:10.1534/g3.113.007294)
Supplement: Supporting Information [file supp_g3.113.007294_TableS1.pdf]

Table S1 The donor line/lines of each line in the Reopened panel.

Reopened lines	Donor lines
C113.004	Chevron
C119.002	Chevron
FEG59.09	Ac Oxbow
FEG60.27	BT463
FEG61.37	Clho6613
FEG63.16	Chevron
FEG63.56	Chevron
FEG65.02	Zhedar1
FEG66.05	Zhedar1
FEG66.08	Zhedar1
FEG66.21	Zhedar1
FEG66.31	Zhedar1
FEG67.12	Frederickson
FEG69.24	PFC88209
FEG69.38	PFC88209
FEG73.13	Hor211
FEG73.49	Hor211
FEG74.18	Hor211
FEG74.19	Hor211
FEG75.39	Hor211
FEG80.06	Zhedar1
FEG80.53	Zhedar1
FEG81.58	Harrington
FEG81.60	Harrington
FEG82.16	Chevron
FEG86.03	Hor211
FEG86.53	Hor211
FEG88.73	Atahualpa, Zhedar1
FEG88.87	Atahualpa, Zhedar1
FEG89.73	Hor211
FEG90.31	Zhedar1, Atahualpa
FEG90.35	Zhedar1, Atahualpa
FEG91.28	PFC88209, Frederickson
FEG93.12	Frederickson
FEG93.36	Frederickson
FEG94.20	Zhedar1

FEG94.41	Zhedar1
FEG96.06	Ac Oxbow
FEG96.55	Ac Oxbow
FEG97.14	Ac Oxbow
FEG97.44	Ac Oxbow
FEG98.53	PFC88209
FEG99.10	Ac Oxbow
FEG99.51	Ac Oxbow
FEG100.17	Zhedar1
FEG100.33	Zhedar1
FEG100.41	Zhedar1
FEG100.47	Zhedar1
FEG103.44	Ac Oxbow, Harrington
FEG103.45	Ac Oxbow, Harrington
FEG104.63	Zhedar1
FEG104.89	Zhedar1
FEG105.33	PFC88209
FEG105.59	PFC88209
FEG109.13	Ac Oxbow
FEG109.44	Ac Oxbow
FEG109.54	Ac Oxbow
FEG111.10	Ac Oxbow, Zhedar1
FEG111.13	Ac Oxbow, Zhedar1
FEG111.24	Ac Oxbow, Zhedar1
FEG112.14	Ac Oxbow, Atahualpa
FEG113.85	Ac Oxbow, Zhedar1
FEG114.33	Clho6613
FEG116.05	Zhedar1
FEG117.24	Zhedar1
FEG118.05	PFC88209
FEG118.41	PFC88209
FEG118.69	PFC88209
FEG121.03	Zhedar1, Ac Oxbow
FEG121.16	Zhedar1, Ac Oxbow
FEG121.29	Zhedar1, Ac Oxbow
FEG121.43	Zhedar1, Ac Oxbow
FEG122.36	Hor211, PFC88209
FEG122.50	Hor211, PFC88209
FEG122.92	Hor211, PFC88209

FEG124.35	PFC88209
FEG125.46	Zhedar1
FEG125.69	Zhedar1
FEG126.08	Zhedar1
FEG126.14	Zhedar1
FEG129.41	Frederickson
FEG129.60	Frederickson
FEG132.05	Zhedar1, Frederickson
FEG132.63	Zhedar1, Frederickson
FEG138.08	Zhedar1, Hor211
FEG138.27	Zhedar1, Hor211
FEG141.18	Ac Oxbow
FEG141.20	Ac Oxbow
FEG142.13	Zhedar1, Hor211
FEG142.28	Zhedar1, Hor211
FEG142.55	Zhedar1, Hor211
FEG144.21	Ac Oxbow, Hor211
FEG144.27	Ac Oxbow, Hor211
FEG144.68	Ac Oxbow, Hor211
FEG146.09	Frederickson
FEG146.46	Frederickson
FEG146.68	Frederickson
FEG147.03	Zhedar1, Atahualpa
FEG147.14	Zhedar1, Atahualpa
FEG147.63	Zhedar1, Atahualpa
FEG148.22	Ac Oxbow
FEG148.56	Ac Oxbow
FEG149.18	ND20407
FEG149.65	ND20407
FEG150.42	ND20493
FEG150.49	ND20493
FEG153.22	Zhedar1
FEG155.07	Ac Oxbow
FEG156.09	Zhedar1
FEG161.03	Ac Oxbow
FEG162.22	Ac Oxbow
FEG163.21	Zhedar1
FEG164.33	Hor211, PFC88209
FEG166.38	Zhedar1

FEG168.09	Comp351
FEG169.47	Hor211
FEG170.07	Hor211
FEG172.40	Hor211
FEG175.57	Zhedar1, Hor211
