# Supplementary material for: Comparative Analyses Identify the Contributions of Exotic Donors to Disease Resistance in a Barley Experimental Population
Source: G3 (Bethesda). 2013 Nov 1;3(11):1945–53. doi: 10.1534/g3.113.007294 (PMC3815057; doi:10.1534/g3.113.007294)
Supplement: Supporting Information [file supp_g3.113.007294_TableS2.pdf]

Table S2 The observed pairwise diversity (scaled by the number of segregating sites) for each linkage group and the median of simulated pairwise diversity in the Ancestral panel, Closed, and Reopened panel.

LG	Ancestral panel	Closed panel	Reopened panel
1H	0.049	0.002	0.004
2H	0.060	0.004	0.004
3H	0.072	0.001	0.005
4H	0.057	0.001	0.005
5H	0.068	0.004	0.007
6H	0.059	0.007	0.011
7H	0.044	0.002	0.002
Simulated	0.054	0.007	0.014
