# Supplementary material for: Comparative Analyses Identify the Contributions of Exotic Donors to Disease Resistance in a Barley Experimental Population
Source: G3 (Bethesda). 2013 Nov 1;3(11):1945–53. doi: 10.1534/g3.113.007294 (PMC3815057; doi:10.1534/g3.113.007294)
Supplement: Supporting Information [file supp_g3.113.007294_TableS3.pdf]

Table S3 Markers from previous studies that are within or flanking (~5 cM) the high F_{ST} blocks and their estimated positions.

Linkage group	Marker	Position (cM)
2H	ABC252	141.89 – 142.42
	CDO373	
	F3hA	
	MWG5208	
	pKABA1	
	Ebmc0415	142.42 – 143.72
	Cnx1	
	BCD135	146.05 – 147.93
	Gln2	
	KG004.1	
	KG004.2	
	ABC157	148.58 – 150.55
	Zeo1	150.55 – 152.64
4H	HVM40	19.27 – 20.26
	CDO669A	20.68 – 21.33
	Ole1	24.49 – 25.23
	BCD402B	26.47
	CDO542	29.08 – 29.49
	DsT-29	
	CDO122	30.10
	BCD351D	30.10 – 31.41
	INT-C	30.37
	MWG635A	31.41 – 32.62
	BCD265B	34.92 – 37.54
	BCD808B	
5H	Scssr05939	94.25 – 94.66
	ksuD17	65.89 – 69.87
	G57	
	ABC163	71.52 – 72.17
	ABG379	
	Bmac0218C	73.18 – 73.98
	ABG388	74.60 – 75.34
	CDO507	
	ABC175	77.82 – 78.46
	RZ323	

6H	ksuA3D	79.34 – 80.13
	ABC1708	
	Scsnp21226	81.05 – 82.53
	MWG820	83.71 – 85.17
	cMWG684D	88.08 – 88.78
	MWG514	136.17 – 137.84
	MWG798A	
	ABG725	138.49 – 140.92
	DAK213C	
	DsT-71	
