# Supplementary material for: Comparative Analyses Identify the Contributions of Exotic Donors to Disease Resistance in a Barley Experimental Population
Source: G3 (Bethesda). 2013 Nov 1;3(11):1945–53. doi: 10.1534/g3.113.007294 (PMC3815057; doi:10.1534/g3.113.007294)
Supplement: Supporting Information [file supp_g3.113.007294_TableS4.pdf]

Table S4 BOPA, POPA and SCRI SNPs within genes of known function in the high F_{ST} blocks and their respective gene products.

LG	SNP	GenBankID	cM	Silent	Gene	Product
2H	SCRI_RS_173017	NM_001073041	139.9	No	Os12g0256900	hypothetic protein
	11_10446	XM_003560174	140.69	No	LOC100837523	serine carboxypeptidase-like
	11_20480	AY162186	140.69	Yes	exin1	Extracellular invertase
	SCRI_RS_15119	DQ163025	141.5	Yes	VTE5	phytol kinase
	11_21459	AM039897	143.18	No	ahh1	S-adenosyl-L-homocysteine hydrolase
	2_1484	AB058924	143.71	Yes	HvPKABA1	protein kinase HvPKABA1
	11_10656	XM_003580700	145.69	Yes	LOC100826196	U3 small nucleolar ribonucleoprotein protein IMP3-like
	11_10383	AY136627	147.37	Yes	Ha1	plasma membrane P-type proton pump ATPase
	12_30942	GQ169685	147.37	Yes	GS2	plastid glutamine synthetase 2
4H	11_20422	XM_003560743	28	Yes	LOC100820964	microsomal glutathione S-transferase 3-like isoform 1
	SCRI_RS_157832	XM_003560569	30	Yes	LOC100840876	vam6/Vps39-like protein-like
6H	SCRI_RS_143317	XM_003570599	72.9	No	LOC100831957	RINT1-like protein-like
	SCRI_RS_206976	XM_003570630	74.6	No	LOC100841641	microtubule-associated protein TORTIFOLIA1-like
